# Large Language Model Assistant for Emergency Department Discharge Documentation

**DOI:** 10.1001/jamanetworkopen.2025.38427

**Published:** 2025-10-21

**Authors:** Ji Woo Song, Junseong Park, Ji Hoon Kim, Seng Chan You

**Affiliations:** 1 Yonsei University College of Medicine, Seoul, South Korea; 2 Department of Emergency Medicine, Yonsei University College of Medicine, Seoul, South Korea; 3 Institute for Innovation in Digital Health, Yonsei University, Seoul, South Korea; 4 Department of Biomedical Systems Informatics, Yonsei University College of Medicine, Seoul, South Korea

## Abstract

**Importance:**

Emergency department (ED) discharge documentation is time-consuming and often incomplete.

**Objective:**

To develop a large language model (LLM) assistant that generates ED discharge notes and to evaluate its effectiveness on documentation quality and workflow efficiency.

**Design, Setting, and Participants:**

This comparative effectiveness study, which was conducted at a 2400-bed tertiary care hospital in South Korea, consisted of 2 primary phases: a development phase and sequential validation of the LLM assistant. In the randomized sequential prospective validation, 6 emergency physicians first wrote discharge notes manually (session 1), then edited LLM-generated drafts after a 1-hour washout period (session 2). Three independent physicians evaluated 300 note sets (each containing a manual note, an LLM draft, and an LLM-assisted note). For model development and validation, patient records from ED visits between September 1, 2022, and August 31, 2023, were used. The inclusion criteria encompassed adult patients (aged ≥17 years) and pediatric patients with nondisease conditions (eg, trauma, poisoning, or burns). Emergency physicians selected 592 representative cases for training and 50 for validation.

**Exposure:**

A commercially available text generation transformer model was used as a core LLM, fine-tuned using the 592 training cases. Two distinct processing pipelines were implemented within the LLM assistant due to different input data: (1) for patients managed solely by emergency physicians, using the ED initial record and prescription list, and (2) for those requiring specialty consultations, using the ED initial record and consultation request form.

**Main Outcomes and Measures:**

Quality of notes using 4C metrics (completeness, correctness, conciseness, and clinical utility) on a Likert scale ranging from 1 to 5 and time taken to complete the notes manually and with the LLM assistant.

**Results:**

Of the 50 test cases, the mean (SD) patient age was 57.7 (23.1) years, and 28 patients (56%) were female. LLM-assisted notes achieved higher scores than manual notes in completeness (4.23 [95% CI, 4.17-4.28] vs 4.03 [95% CI, 3.96-4.09]), correctness (4.38 [95% CI, 4.33-4.42] vs 4.20 [95% CI, 4.14-4.26]), conciseness (4.23 [95% CI, 4.18-4.28] vs 4.11 [95% CI, 4.05-4.17]), and clinical utility (4.17 [95% CI, 4.11-4.23] vs 3.85 [95% CI, 3.78-3.91]) (all *P* < .001). When compared with LLM drafts, LLM-assisted notes excelled in conciseness (4.23 vs 3.98 [95% CI, 3.91-4.04]; *P* < .001) and maintained equivalent clinical utility (4.17 vs 4.16 [95% CI, 4.11-4.21]; *P* > .99), but scored lower in completeness (4.23 vs 4.34 [95% CI, 4.29-4.39]; *P* = .001) and correctness (4.38 vs 4.45 [95% CI, 4.41-4.49]; *P* < .001). The median documentation time per note dropped from 69.5 (95% CI, 65.5-78.0) seconds for manual notes to 32.0 (95% CI, 29.5-36.0) seconds for LLM-assisted notes (*P* < .001).

**Conclusion:**

In this comparative effectiveness study, use of an on-site LLM assistant was associated with reduced writing time for ED discharge notes compared with manual note-taking, without compromising documentation quality, representing a critical advancement in the use of artificial intelligence for clinical practice.

## Key Points

**Question:**

Can an on-site large language model (LLM) assistant help emergency physicians write discharge notes faster without compromising the quality of the notes?

**Findings:**

In this comparative effectiveness study including 300 manual notes, 300 LLM drafts, and 300 LLM-assisted notes from 50 cases, the LLM-assisted notes were more complete, correct, concise, and clinically useful than manual notes. The median writing time decreased from 69.5 to 32.0 seconds with LLM assistance.

**Meaning:**

These findings suggest that LLM assistance is a promising solution for generating high-quality discharge notes with lesser burden on emergency physicians.

## Teaser

This comparative effectiveness study investigates a large language model designed to assist emergency department physicians with writing discharge notes and evaluates its effectiveness on documentation quality and workflow efficiency compared with manual note writing.

## Introduction

Discharge notes in the emergency department (ED) are crucial for ensuring high-quality patient care by documenting treatment details, supporting continuity for returning patients, and facilitating smooth transitions to community care.^1,2^ However, in the chaotic and urgent environment of the ED, creating high-quality discharge notes is often challenging and time-consuming, leading to delayed, incomplete, or missing documentation.^3,4^ Since poor-quality discharge notes may contribute to prescription inaccuracies, delayed follow-ups, and increased readmission rates,^1,2^ leveraging large language model (LLMs) to assist with discharge documentation has recently gained interest.^5,6,7^

Previous studies exploring LLM-based discharge note generation have primarily relied on proprietary models,^8^ which could breach data security policies in a real clinical setting.^9,10,11,12^ While some researchers have attempted to address these concerns by using open-source LLMs or deploying proprietary models within secure private cloud environments, these solutions often demonstrated limited performance in real clinical settings, particularly in terms of accuracy and contextual understanding.^13,14^ Furthermore, the computational resources required for larger models posed practical challenges for hospital-wide deployment. To address these limitations, we developed the Your Knowledgeable Navigator of Treatment (Y-KNOT) project, focusing on creating an efficient, on-site LLM-based assistant using a lightweight architecture that balances performance with computational efficiency. The project’s first implementation, Y-KNOT ED discharge note generation assistant (Y-KNOT-EDN), specifically targets ED documentation workflows by processing structured clinical information from the electronic health record (EHR). This study assessed changes in documentation quality and patterns based on Y-KNOT-EDN use within a virtual EHR environment to verify its effectiveness and safety.

## Methods

### Development of Y-KNOT-EDN

This comparative effectiveness study was conducted at an urban academic 2400-bed tertiary care hospital in Seoul, Korea. The level 1 ED at this hospital handles approximately 60 000 patient visits annually and is responsible for managing severe emergency cases in the northwestern region of Seoul. In this ED, approximately 70% of patients are discharged by emergency physicians, and their discharge notes are typically written by emergency medicine physicians or residents. The Institutional Review Board of Yonsei University approved the study and granted a waiver of informed consent owing to the use of retrospective data. The study design and reporting adhered to the International Society for Pharmacoeconomics and Outcomes Research (ISPOR) reporting guideline for comparative effectiveness studies and the Transparent Reporting of a Multivariable Prediction Model for Individual Prognosis or Diagnosis (TRIPOD) LLM reporting guideline.^15^

The technical details of Y-KNOT-med-base, the core model underlying Y-KNOT-EDN, including its model architecture and pretraining process, are described previously.^16^ In brief, we developed a lightweight LLM based on a commercially available text generation transformer model (Llama3-8B; Meta), further pretrained it with 9.0 GB of general data and 90.4 GB of medical knowledge data, and fine-tuned it with actual ED discharge cases using instruction tuning (eMethods 1 in Supplement 1). For instruction tuning, we retrieved data for patients visiting the ED between September 1, 2022, and August 31, 2023. Eligible records required complete ED and discharge documentation finalized within 48 hours. The study included adult patients (aged ≥17 years) and pediatric patients with nondisease conditions (eg, trauma, poisoning, or burn) typically managed by emergency physicians. Among pediatric patients, those who visited the pediatric ED under a pediatrician and were discharged thereafter were excluded. Deceased patients were excluded. Data were stratified to ensure an even monthly distribution, yielding 2028 cases. From these cases, 2 emergency physicians—a board-certified emergency physician (J.H.K.) and a fourth-year ED resident (J.P.)—selected 592 representative cases for instruction tuning the LLM. The selection criteria emphasized common ED presentations, varying complexity levels, and completeness of the clinical records to ensure realistic ED discharge scenarios (eFigure 1 in Supplement 1). The emergency physicians reviewed each patient’s full medical record to identify reliable sources for 6 core components in each discharge note—medical history, reason for visit, test orders, test results, specialty consultation details, and future plans—to ensure that Y-KNOT-EDN produces discharge notes as similar to those produced by emergency physicians as possible. While the primary diagnosis is not directly generated by Y-KNOT-EDN, it is captured within the EHR system’s structured template, ensuring that the narrative focuses on comprehensive discharge details while the diagnosis is documented elsewhere as structured data (eFigure 2 in Supplement 1).

**Figure 1.**
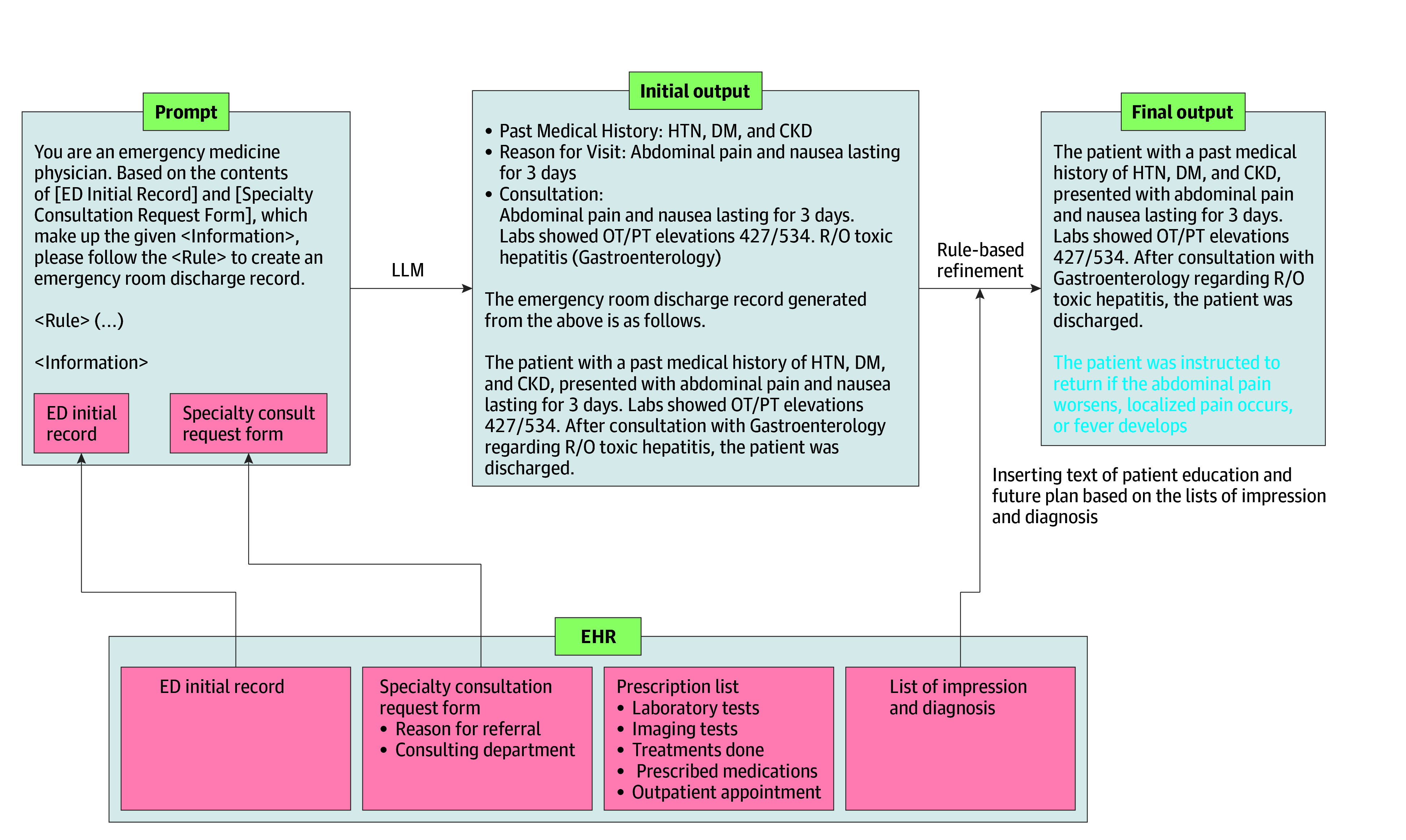
Large Language Model (LLM) Pipeline for Cases With Specialty Consultations Involved For cases involving specialty consultations, the system uses the emergency department (ED) initial record and specialty consultation request form to generate a comprehensive draft. Rule-based postprocessing adds standardized patient education statements, ensuring the final summaries align with ED documentation standards. Blue text indicates the inserted patient education content generated during this refinement step. CKD indicates chronic kidney disease; DM, diabetes; EHR, electronic health record; HTN, hypertension; OT/PT, oxaloacetic transaminase and/or pyruvic transsaminase; R/O, rule out.

**Figure 2.**
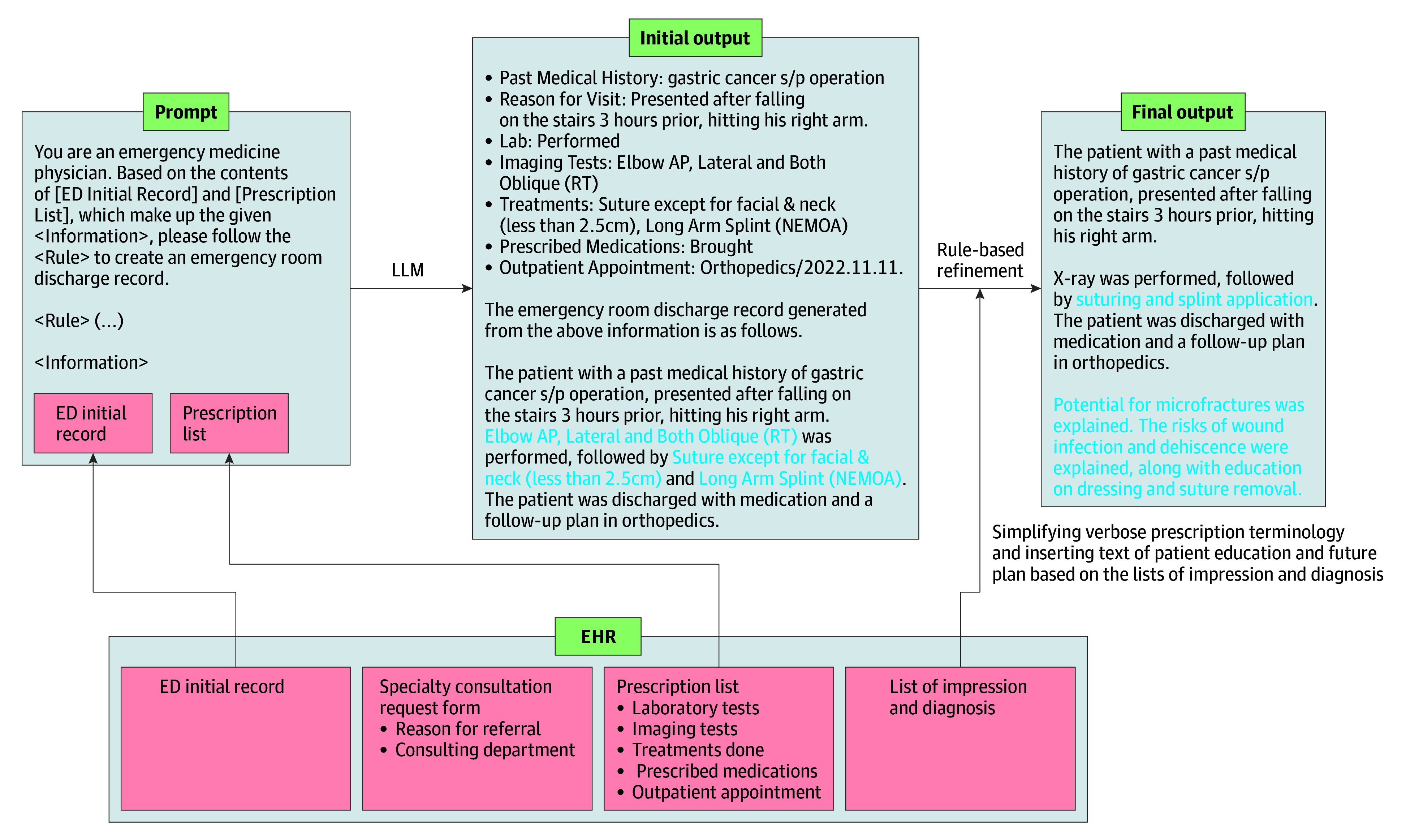
Large Language Model (LLM) Pipeline for Cases Managed by Emergency Department (ED) Physicians Alone In cases without specialty consultations, the system references the ED initial record and prescription list to create a draft discharge note. Rule-based postprocessing adds standardized patient education statements. Rule-based postprocessing simplifies prescription terminologies and adds standardized patient education statements, ensuring the final summaries align with ED documentation standards. Blue text indicates these refined components, including simplified terminology and inserted patient education content. AP indicates anterior-posterior; EHR, electronic health record; RT, right; s/p, status post.

Only consistently available and reliable data sources were selected as input for the LLM. Based on the 2 possible clinical pathways in the ED—patients requiring specialty consultations or those managed solely by emergency physicians—we developed 2 distinct processing pipelines. In cases requiring 1 or more specialty consultations, the system drew on the ED initial record and specialty consultation request form to capture a concise yet accurate outline of the patient’s history, examination findings, and basic investigations, key details that emergency physicians typically document when requesting a specialty consultation (Figure 1). By contrast, if managed only by emergency physicians, Y-KNOT-EDN references only the ED initial record and the prescription list (Figure 2). After either pipeline assembles the relevant data, a predefined set of instruction prompts are given to the LLM, which then produces an initial draft. Finally, rule-based mechanisms insert standardized patient education statements and streamline verbose prescription terminology, ensuring the final drafts align with the clinical and legal requirements of discharge notes. Two physicians (J.P. and a pediatrician with 27 years of clinical experience) evaluated the output of LLM throughout the instruction-tuning process. The example of the overall structure of the instruction prompt and the output of LLM are demonstrated in Figure 1 and Figure 2. More detailed selection and curation process of representative cases for instruction tuning can be found in eMethods 2 in Supplement 1.

### In Silico Randomized Sequential Evaluation of Y-KNOT-EDN

After excluding this instruction-tuning dataset, 50 independent cases were selected for testing using stratified sampling to ensure representation across consultation complexity levels: 20 with no specialty consultations, 20 requiring a single specialty consultation, and 10 requiring multiple consultations. All discharge reports contained a mixture of Korean and English entries.

The in silico randomized sequential evaluation was conducted in 2 steps, illustrated in eFigure 1 in Supplement 1. First, 6 emergency physicians were asked to write ED discharge notes of 50 representative test cases without and with the assistance of Y-KNOT-EDN in the virtual EHR environment. In session 1, physicians manually wrote discharge notes in the virtual EHR interface, resulting in 300 manual notes. After a 1-hour washout period, session 2 featured the same cases in a randomized order, with LLM drafts preloaded into the EHR interface for direct editing, resulting in 300 LLM-assisted notes. The time taken to complete the notes was recorded in both sessions, and a brief survey about the user experience of Y-KNOT-EDN was conducted. Subsequently, 3 attending emergency physicians with 6, 8, and 9 years of ED experience, all of whom were entirely independent from the 6 physicians who generated the manual and LLM-assisted notes, performed a blinded assessment of the 3 note types (manual note, LLM draft, and LLM-assisted note). We used 4C metrics (completeness, correctness, conciseness, and clinical utility)^17,18^ to capture crucial aspects of clinical document summarization, whose definitions are listed in eTable 1 in Supplement 1. A customized interface was used to display the 3 note types simultaneously in random order (eFigure 3 in Supplement 1), all of which were rated on a Likert scale ranging from 1 to 5. The 3 note types for each case were also presented in random order and labeled only as A, B, and C, ensuring that evaluators could not identify which note type they were assessing based on presentation order alone. Additionally, a sensitivity analysis was conducted because the same 50 LLM drafts were evaluated repeatedly 6 times, which might have introduced bias. To eliminate the possibility of the evaluators’ recognition of the LLM drafts during evaluation, a separate analysis was performed using only the first evaluation result for each 50 cases. We also audited 50 LLM drafts for omissions and confabulations; omissions were cross-checked against the 6 physicians’ manual notes, and confabulations were checked for correction in each physician’s LLM-assisted note.

### Similarity Analysis

To quantitatively evaluate whether each clinician’s LLM-assisted note more closely resembled their manual note or the LLM draft, we used the LLM-assisted note as the reference text and calculated textual and semantic similarities to the manual note and the LLM draft (eFigure 1 in Supplement 1). For textual similarity, the Recall-Oriented Understudy for Gisting Evaluation (ROUGE-L) metric was used. ROUGE-L focuses on the exact wording and measures how much the n-grams (sequences of words) in the generated text overlap with those in the reference text; scores range from 0 to 1, with lower scores indicating lower overlap between generated and reference text and higher scores indicating greater overlap. In contrast, for semantic similarity, BERTScore was used to evaluate the similarity of meaning by analyzing the contextual embeddings of the texts, capturing how similar the underlying ideas are, rather than just the specific words used. BERTScores range from 0 to 1, with lower scores indicating lower semantic similarity and higher scores indicating greater semantic similarity between generated and reference text.

### Statistical Analysis

For clinical analysis, a Friedman test was conducted on the 3 note types. If significant differences emerged, pairwise comparisons were performed using the Wilcoxon signed rank test with Bonferroni correction. Pairwise effect sizes were quantified by Hedges *g*. Interrater reliability was then assessed, given the subjective nature of ratings; to align rating distributions, each rater’s scores were *z*-score normalized within each metric. After normalization, we calculated 2-way random-effects, consistency, multiple-rater intraclass correlation coefficients using Pingouin, version 0.5.5 (Python Software Foundation).

Writing time for each note was also analyzed nonparametrically. Within-encounter differences between manual notes (session 1) and LLM-assisted notes (session 2) were summarized by the paired Hodges-Lehmann (H-L) median difference. Writing-time reduction attributable to LLM assistance was additionally modeled with a crossed random-effects log-normal mixed model containing fixed effects for LLM use and 3-level complexity, plus random intercepts for patient-case and physician-writer. For similarity analysis, the Shapiro-Wilk test was used to check normality. If scores were normally distributed (*P* > .05), paired *t* tests were used; otherwise, Wilcoxon signed rank tests were applied. All 95% CIs used 1000-resample percentile bootstraps, and all statistical significance was 2 sided.

## Results

### Overall Results

**Figure 3.**
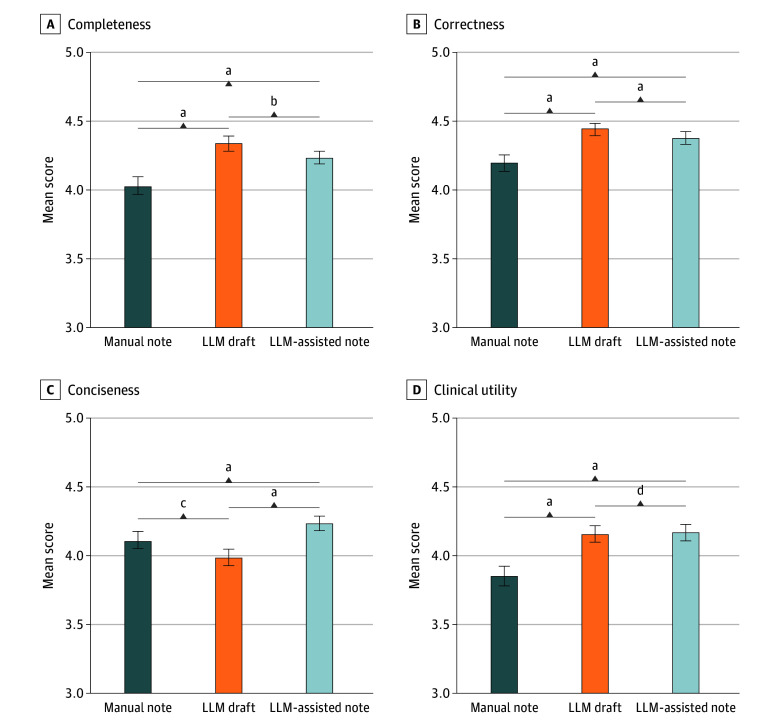
Main Results Based on 4C (Completeness, Correctness, Conciseness, and Clinical Utility) Metrics Error bars indicate 95% bootstrap CIs; exact Bonferroni-adjusted *P* values are annotated between paired comparisons. The y-axes are truncated to the clinically relevant range (3.0-5.0). ^a^*P* < .001. ^b^*P* = .001. ^c^*P* = .004. ^d^*P* > .99.

Of the 50 test cases, the mean (SD) age was 57.7 (23.1) years; 28 patients (56%) were female and 22 (44%) were male. The ratio of trauma to medical cases was 17:33, and the Korean Triage and Acuity Scale distribution consisted of 5 patients at level 2, 15 patients at level 3, 23 patients at level 4, and 7 patients at level 5. The results of clinical analyses based on the 4C metrics are shown in Figure 3. The mean LLM draft score was higher in completeness (4.34; 95% CI, 4.29-4.39) and correctness (4.45; 95% CI, 4.41-4.49) compared with both the LLM-assisted notes (4.23 [95% CI, 4.17-4.28; *P* = .001] in completeness; 4.38 [95% CI, 4.33-4.42; *P* < .001] in correctness) and the manual notes (4.03 [95% CI, 3.96-4.09; *P* < .001] in completeness; 4.20 [95% CI, 4.14-4.25; *P* < .001] in correctness). The mean score for the LLM-assisted note also outperformed that for the manual note in both metrics (both *P* < .001). For conciseness, the LLM-assisted note (4.23; 95% CI, 4.18-4.28) had the best mean score, outperforming both the manual note (4.11; 95% CI, 4.05-4.17; *P* < .001) and LLM draft (3.98; 95% CI, 3.91-4.04; *P* < .001). The manual note was also more concise than the LLM draft (*P* = .004). In terms of clinical utility, the LLM draft (4.16; 95% CI, 4.11-4.21) and LLM-assisted note (4.17; 95% CI, 4.11-4.23) were similarly useful (*P* > .99), and both were more useful than the manual note (3.85; 95% CI, 3.78-3.91; both *P* < .001). Detailed results—including 95% CIs, Hedges *g*, and H-L estimates—are presented in eTable 2 in Supplement 1.

The analysis revealed intraclass correlation coefficient values of 0.58 (95% CI, 0.50-0.64) for completeness, 0.39 (95% CI, 0.27-0.47) for correctness, 0.62 (95% CI, 0.55-0.68) for conciseness, and 0.55 (95% CI, 0.48-0.62) for clinical utility. eFigure 4 in Supplement 1 displays the pairwise 5 × 5 rating heat maps that show where evaluators’ scores coincide and where they diverge. These results indicate moderate reliability for conciseness and completeness, while showing fair agreement for clinical utility and relatively lower reliability for correctness among the raters.

### Subgroup Results Based on Consultation Complexity

For cases managed solely by emergency physicians, the LLM-assisted note outperformed both the manual note and the initial LLM draft in clinical utility. Interestingly, for cases involving 1 or more specialty consultations, the unedited LLM draft proved more clinically useful than both the manual note and the LLM-assisted note. The detailed results are presented in eFigure 5 and eTable 3 in Supplement 1.

### Sensitivity Analysis

A sensitivity analysis examining only the first 50 cases per evaluator (total of 150 cases) demonstrated the LLM-assisted note’s superiority to the manual note in clinical utility (4.43 [95% CI, 4.30-4.55] vs 4.17 [95% CI, 4.04-4.31]; *P* = .03) and noninferiority to the manual note in completeness (4.43 [95% CI, 4.30-4.55] vs 4.20 [95% CI, 4.05-4.35]; *P* = .06), correctness (4.75 [95% CI, 4.63-4.85] vs 4.69 [95% CI, 4.56-4.79]; *P* = .96), and conciseness (4.36 [95% CI, 4.21-4.49] vs 4.27 [95% CI, 4.10-4.44]; *P* > .99) (eTable 4 in Supplement 1). The LLM-assisted note scored higher than the LLM draft in conciseness, while the other metrics showed no significant difference.

### Similarity Analysis: ROUGE and BERTScore

**Figure 4.**
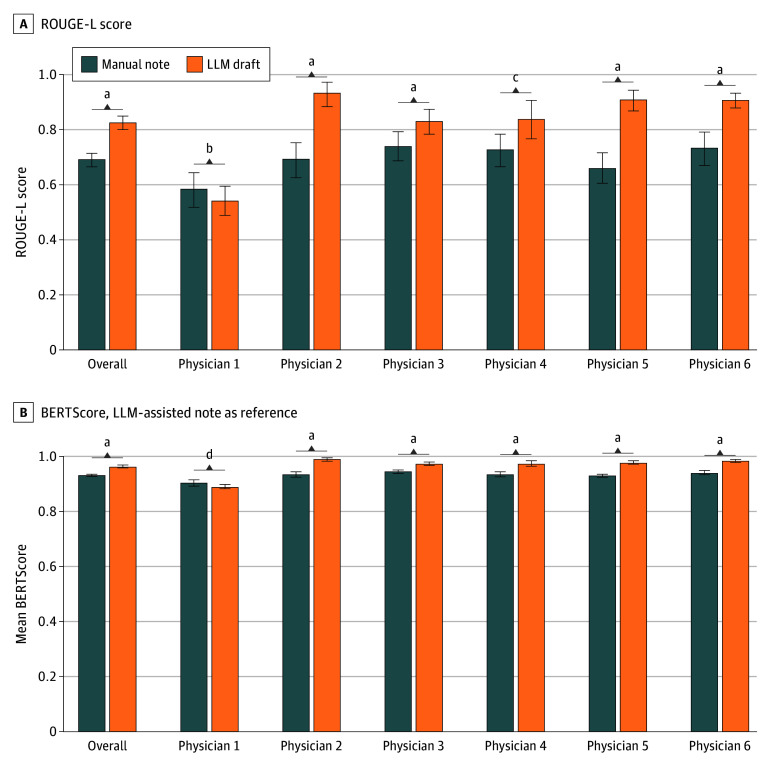
Similarity Analysis Results Bars depict mean scores of Recall-Oriented Understudy for Gisting Evaluation–L (ROUGE-L) or BERTScore of manual notes and large language model (LLM) drafts with 95% bootstrap CIs. LLM-assisted notes were the reference texts. ^a^*P* < .001. ^b^*P* = .40. ^c^*P* = .009. ^d^*P* = .08.

Figure 4 presents the ROUGE-L scores and BERTScore comparing LLM-assisted notes with both manual notes and LLM drafts. On the one hand, the mean ROUGE-L score for LLM-assisted notes vs manual notes was 0.69 (95% CI, 0.67-0.72), which was lower than the ROUGE-L score for LLM-assisted notes vs LLM drafts (0.83 [95% CI, 0.80-0.85]), showing a difference of 0.14 (95% CI, 0.11-0.17; *P* < .001). This finding was particularly pronounced for physicians 2 to 6, while for physician 1, the manual notes were not statistically different but were slightly more textually similar to the LLM-assisted notes than to the LLM drafts (0.59 [95% CI, 0.52-0.65] vs 0.54 [95% CI, 0.49-0.60]; *P* = .40). On the other hand, the BERTScore indicated strong semantic similarity between LLM-assisted notes and both manual notes and LLM drafts. Overall, the BERTScore for LLM-assisted notes vs manual notes was 0.93 (95% CI, 0.93-0.94), lower than that for LLM-assisted notes vs LLM drafts (0.97 [95% CI, 0.96-0.97]), with a difference of 0.04 (95% CI, 0.03-0.04; *P* < .001). Both values indicate high semantic similarity. This pattern, with the LLM draft being slightly more semantically aligned with the LLM-assisted note than the manual note, was evident among physicians 2 to 6. For physician 1, however, the difference was not statistically significant, and the manual notes were marginally more semantically similar to the LLM-assisted notes than to the LLM drafts (0.90 [95% CI, 0.90-0.91] vs 0.89 [95% CI, 0.88-0.90]; *P* = .08). Detailed results of entire ROUGE scores and BERTScore are available in eTable 5 in Supplement 1.

### Comparison of Writing Time of the Manual Note and the LLM-Assisted Note

**Figure 5.**
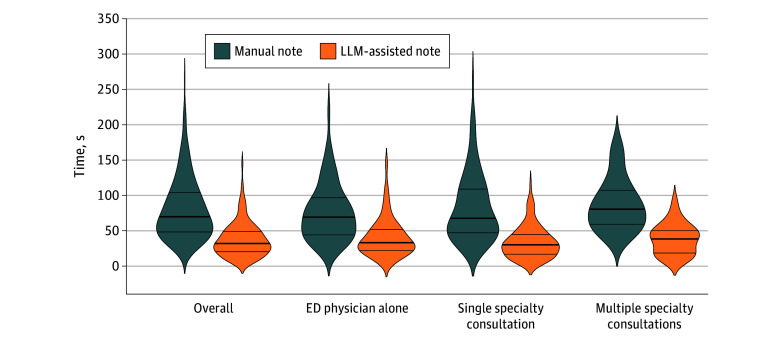
Documentation of Time Comparison Between Manual and Large Language Model (LLM)–Assisted Notes Across Complexity Categories Violin plots comparing documentation times for manual vs LLM-assisted discharge notes across overall encounters and by complexity category. Inner dashed lines mark quartiles. The median differences between groups were all significant at *P* < .001. ED indicates emergency department.

Median-based estimates showed a consistent reduction in writing time when the LLM assistant was used (Figure 5 and eTable 6 in Supplement 1). Overall, the median time decreased from 69.5 (95% CI, 65.5-78.0) seconds for manual notes to 32.0 (95% CI, 29.5-36.0) seconds with LLM support, an H-L median reduction of 35.0 (95% CI, 29.5-41.5) seconds. In notes written by the ED physician alone, the medians decreased from 69.0 (95% CI, 58.0-78.5) to 33.0 (95% CI, 28.0-39.0) seconds, with an H-L reduction of 24.0 (95% CI, 20.0-32.0) seconds. For single-specialty consultations, the medians decreased from 67.5 (95% CI, 60.0-83.0) to 30.0 (95% CI, 26.0-34.0) seconds, with an H-L reduction of 43.0 (95% CI, 35.0-52.0) seconds. For multiple-specialty consultations, writing time declined from 80.5 (95% CI, 68.5-92.5) to 38.5 (95% CI, 28.0-46.0) seconds, giving an H-L reduction of 48.5 (95% CI, 31.0-55.0) seconds. All bootstrap pairwise median comparisons were significant at *P* < .001.

Additionally, the mixed-model analysis confirmed that the writing time was significantly shorter (time ratio, 0.43; 95% CI, 0.39-0.47; *P* < .001) with LLM assistance. Stratified analyses based on case-complexity demonstrated consistent efficiency gains (eFigure 6 in Supplement 1).

### User Experience of Y-KNOT-EDN

A brief survey about the user experience of Y-KNOT-EDN was conducted with the 6 physicians who wrote discharge notes with Y-KNOT-EDN. They highly rated Y-KNOT-EDN’s consistency, coherence, and time-saving benefits but expressed moderate concerns about patient safety and the need for revision before finalizing notes (eTable 7 in Supplement 1).

### Error Audit of LLM Drafts

In a targeted audit of the 50 LLM drafts, 6 omission cases and 1 confabulation case were identified (examples summarized in eTable 8 in Supplement 1). To contextualize the omissions, we examined the corresponding manual notes for each omission case across all 6 physicians (6 cases × 6 physicians = 36 notes): 21 of 36 (58%) omitted the same item, suggesting low salience rather than safety-critical details. The single confabulation documented unperformed procedures (splint application, dressing) and was removed in 5 of 6 LLM-assisted notes—by all except the least-experienced physician—indicating that brief clinician review remains necessary despite high baseline quality.

## Discussion

In this in silico, randomized sequential comparative effectiveness evaluation of an on-site LLM for generating ED discharge documentation, our primary finding was the substantial reduction in documentation time achieved with LLM assistance: physicians’ writing time was significantly shorter with LLM assistance (time ratio, 0.43 [95% CI, 0.39-0.47; *P* < .001]). Additionally, LLM-assisted notes demonstrated better quality compared with manually created notes across completeness, correctness, conciseness, and clinical utility metrics, although the absolute differences were modest. These findings suggest that LLM integration can bring substantial efficiency gains in ED workflows while maintaining documentation quality, representing a clinically meaningful advancement for busy emergency departments.

These results extend previous pilot observations that LLMs can assist with clinical documentation but often lack domain-specific fine-tuning,^14^ may produce hallucinations,^13^ or risk breaching data privacy when using proprietary models.^9,10,12^ Our approach was purposefully designed to overcome these obstacles. First, we built Y-KNOT-EDN on an open-source and lightweight 8B-parameter model, deployed within a secure, on-site environment. This closed-loop architecture avoids potential leakage of sensitive patient data and violation of domestic regulations regarding cross-border data transfer.^19^ Second, we leveraged clinical insights from emergency physicians to systematically identify the essential components and standardize the structure of discharge notes as they are naturally documented in routine clinical practice. Furthermore, we identified reliable and consistent data sources from the EHR while deliberately excluding information likely to cause hallucinations or inconsistencies in LLM outputs. We developed separate pipelines based on case complexity, particularly whether cases required single or multiple specialty consultations, as the availability and nature of clinical information differed substantially between these scenarios. To quantify residual risks after domain-specific fine-tuning, we conducted a 50-case audit of LLM drafts for confabulations and omissions. We found 6 omissions that were context preserving; notably, in 58% of the corresponding physician-written manual notes, the same items were also omitted, suggesting these were low-salience rather than safety-critical details. One confabulation documented procedures not ordered or performed; 5 of 6 physicians—everyone except the least experienced—removed it during editing. Taken together, domain-specific fine-tuning yielded high baseline quality,^19^ but brief clinician inspection remains necessary to capture rare confabulations.^11^

### Strengths and Limitations

A major strength of our study is the systematic, head-to-head comparison of 3 distinct note types—manual, LLM draft, and LLM assisted—using blinded evaluations by independent raters.^7^ By isolating the role of physician edits on the LLM drafts, we identified both the high baseline performance of the tuned model and the variability introduced by user behavior. The similarity analysis showed that, for most physicians, the edited notes more closely resembled the LLM-generated drafts than their own manually written notes. This finding helps explain the significant reduction in completion time: rather than producing entirely new text, physicians primarily refined the existing draft to align with their individual styles, achieving a balance between completeness and conciseness.

Despite these promising results, there are important limitations. First, the fine-tuning and validation of Y-KNOT-EDN were conducted within a single institution, which inherently carries a risk of overfitting to local documentation styles and clinical practices. Second, only 50 carefully selected cases were used for in silico testing; these may not reflect the full range of clinical complexity encountered in a busy urban ED. Third, although we attempted to minimize recall bias by randomizing case order, physicians might still have benefited from earlier exposure to the clinical scenario or improved their efficiency over time. Fourth, we used the 4C metrics, providing a clear, focused evaluation of note quality but still preliminary in scope and validation. More comprehensive, recently developed evaluation frameworks should be incorporated in future work.^20,21^ Fifth, our evaluation relied on 4C metrics and text-similarity metrics, which assess clinical usefulness but do not directly measure patient comprehension or satisfaction. Since prior works show discharge instructions are often hard for patients to understand, randomly sampled lay evaluations of patient comprehension and satisfaction should be performed in future work.^22^ Sixth, interrater reliability for correctness and clinical utility was fair to moderate, highlighting the inherent subjectivity and variability in qualitative assessments of discharge documentation, suggesting the need for a more advanced and rigorous evaluation framework.^23^ Finally, our deliberate decision to exclude certain unreliable or incomplete EHR fields may limit the model’s ability to handle highly complex scenarios.

## Conclusions

In this comparative effectiveness study, the use of an LLM assistant was associated with reduced writing time for ED discharge notes compared with manual note-taking, without compromising documentation quality. By reducing physician workload and enhancing documentation quality, this LLM assistant represents a critical advancement in leveraging artificial intelligence for clinical practice.^12^ Future research will focus on refining its integration with clinical workflows and assessing its long-term impact on patient care and physician well-being.

## Notes

Supplement 1. **eMethods 1.** Model Architecture, Training Process, and Technology Specifications **eMethods 2.** Selection and Curation of Representative Emergency Department Cases for Instruction Tuning **eFigure 1.** Study Design and Evaluation Framework **eFigure 2.** Screenshot of Our Institution’s EHR User Interface **eFigure 3.** Example of the Customized Interface for Blind Evaluation of 3 Kinds of Note Using the 4Cs Metrics **eFigure 4.** Pairwise Rater-Agreement Heat Maps for the 3 Physician Evaluators **eFigure 5.** Subgroup Results Categorized by Complexity of Consultation **eFigure 6.** Expected Writing-Time Ratio (LLM-Assisted or Manual) Estimated From a Crossed Random-Effects Log-Normal Mixed Model **eTable 1.** Definition of 4C Metrics for Qualitative Evaluation of Discharge Notes **eTable 2.** Main Results of Entire Notes **eTable 3.** Subgroup Results Categorized by Consultation Complexity **eTable 4.** Sensitivity Analysis Results **eTable 5.** Textual and Semantic Similarity Captured by ROUGE and BERTScore **eTable 6.** Median Time (Seconds) Required to Write the Manual Note and the LLM Assisted Note: Overall Results and Breakdown by Consultation Complexity and Individual Physician **eTable 7.** User Experience Survey Regarding 12 Aspects of Y-KNOT **eTable 8.** Real Examples of Omissions and Confabulation Identified in the 50-Case Audit of LLM Drafts

Supplement 2.
Data Sharing Statement
