# Supplementary material for: Large Language Model Assistant for Emergency Department Discharge Documentation
Source: JAMA Netw Open. 2025 Oct 21;8(10):e2538427. doi: 10.1001/jamanetworkopen.2025.38427 (PMC12541540; doi:10.1001/jamanetworkopen.2025.38427)
Supplement: Supplement 1. — eMethods 1. Model Architecture, Training Process, and Technology Specifications eMethods 2. Selection and Curation of Representative Emergency Department Cases for Instruction Tuning eFigure 1. Study Design and Evaluation Framework eFigure 2. Screenshot of Our Institution’s EHR User Interface eFigure 3. Example of the Customized Interface for Blind Evaluation of 3 Kinds of Note Using the 4Cs Metrics eFigure 4. Pairwise Rater-Agreement Heat Maps for the 3 Physician Evaluators eFigure 5. Subgroup Results Categorized by Complexity of Consultation eFigure 6. Expected Writing-Time Ratio (LLM-Assisted or Manual) Estimated From a Crossed Random-Effects Log-Normal Mixed Model eTable 1. Definition of 4C Metrics for Qualitative Evaluation of Discharge Notes eTable 2. Main Results of Entire Notes eTable 3. Subgroup Results Categorized by Consultation Complexity eTable 4. Sensitivity Analysis Results eTable 5. Textual and Semantic Similarity Captured by ROUGE and BERTScore eTable 6. Median Time (Seconds) Required to Write the Manual Note and the LLM Assisted Note: Overall Results and Breakdown by Consultation Complexity and Individual Physician eTable 7. User Experience Survey Regarding 12 Aspects of Y-KNOT eTable 8. Real Examples of Omissions and Confabulation Identified in the 50-Case Audit of LLM Drafts [file jamanetwopen-e2538427-s001.pdf]

Supplementary Online Content

Song JW, Park JS, Kim JH, You SC. Large language model assistant for emergency department discharge documentation. *JAMA Netw Open.* 2025;8(10):e2538427.
doi:10.1001/jamanetworkopen.2025.38427

eMethods 1. Model Architecture, Training Process, and Technology Specifications
eMethods 2. Selection and Curation of Representative Emergency Department Cases for Instruction Tuning
eFigure 1. Study Design and Evaluation Framework
eFigure 2. Screenshot of Our Institution's EHR User Interface
eFigure 3. Example of the Customized Interface for Blind Evaluation of 3 Kinds of Note Using the 4Cs Metrics
eFigure 4. Pairwise Rater-Agreement Heat Maps for the 3 Physician Evaluators
eFigure 5. Subgroup Results Categorized by Complexity of Consultation
eFigure 6. Expected Writing-Time Ratio (LLM-Assisted or Manual) Estimated From a Crossed Random-Effects Log-Normal Mixed Model
eTable 1. Definition of 4C Metrics for Qualitative Evaluation of Discharge Notes
eTable 2. Main Results of Entire Notes
eTable 3. Subgroup Results Categorized by Consultation Complexity
eTable 4. Sensitivity Analysis Results
eTable 5. Textual and Semantic Similarity Captured by ROUGE and BERTScore
eTable 6. Median Time (Seconds) Required to Write the Manual Note and the LLM Assisted Note: Overall Results and Breakdown by Consultation Complexity and Individual Physician
eTable 7. User Experience Survey Regarding 12 Aspects of Y-KNOT
eTable 8. Real Examples of Omissions and Confabulation Identified in the 50-Case Audit of LLM Drafts

This supplementary material has been provided by the authors to give readers additional information about their work.

© 2025 Song JW et al. *JAMA Network Open*.

eMethods 1. Model Architecture, Training Process, and Technology Specifications

**Base Model**	Ko-Llama3-Luxia-8B
**Architecture**	Llama3
**Language Coverage**	Bilingual (Korean/English)
**Parameter**	8,030,257,152
**Sequence Length**	32K Token
**Training Method**	FP16, BF16
**GPU**	H100(80GB) * 8EA
**Epochs**	1.0
**Learning Rate**	1e-5
**Batch Size**	128
**Inference**	vLLM, TGI (Acceleration Algorithm)
**Pre-training Data**	Basic Knowledge: 9.0 GB
	Medical Knowledge: 90.4 GB
**Instruction Tuning Data**	592 emergency department cases

eMethods 2. Selection and Curation of Representative Emergency Department Cases for Instruction Tuning

From September 1, 2022, to August 31, 2023, we extracted data for patients who visited the emergency department (ED) of Severance Hospital and were discharged (excluding those who died). We included only patients who had both an ED Initial Record and an ED discharge note, with the discharge note documented within 48 hours. Patients aged 17 or older were classified as adults, and those under 17 were classified as paediatric. Among paediatric patients, those who visited the paediatric ED under a paediatrician and were discharged thereafter were excluded.

One attending emergency medicine physician (JHK) and one fourth-year emergency medicine resident (JSP) then selected patients who presented with non-disease conditions (e.g., trauma, burns, drug intoxication) and were discharged after receiving care from the ED. They grouped cases into the following five categories to ensure diverse coverage of chief complaints and reasons for ED visits, and to evenly distribute cases across the months from January to December:

1. Adult patients with **disease-related conditions** who underwent specialty consultation
2. Adult patients with **disease-related conditions** who did not undergo specialty consultation
3. Adult patients with **non-disease conditions** (e.g., trauma, drug intoxication) who underwent specialty consultation
4. Adult patients with **non-disease conditions** who did not undergo specialty consultation
5. Paediatric patients with **non-disease conditions**

Initially, 2,028 cases were extracted. They reviewed these cases based on typical chief complaints and reasons for ED visits, specialty consultation services, and similarity to actual discharge notes. A final total of 592 cases were then selected.

All relevant records for information sources were used as-is for the input to the model. However, because the actual discharge notes often contain information not available in the input (which could lead to hallucinations), they removed such unavailable details from the original discharge notes. They then cleaned the format to create an output label suitable for model training.

These 592 instruction-tuning cases were used alongside publicly available sources on general text and document summarization for a three-stage training process, enabling multi-task learning.

© 2025 Song JW et al. *JAMA Network Open*.

eFigure 1. Study Design and Evaluation Framework

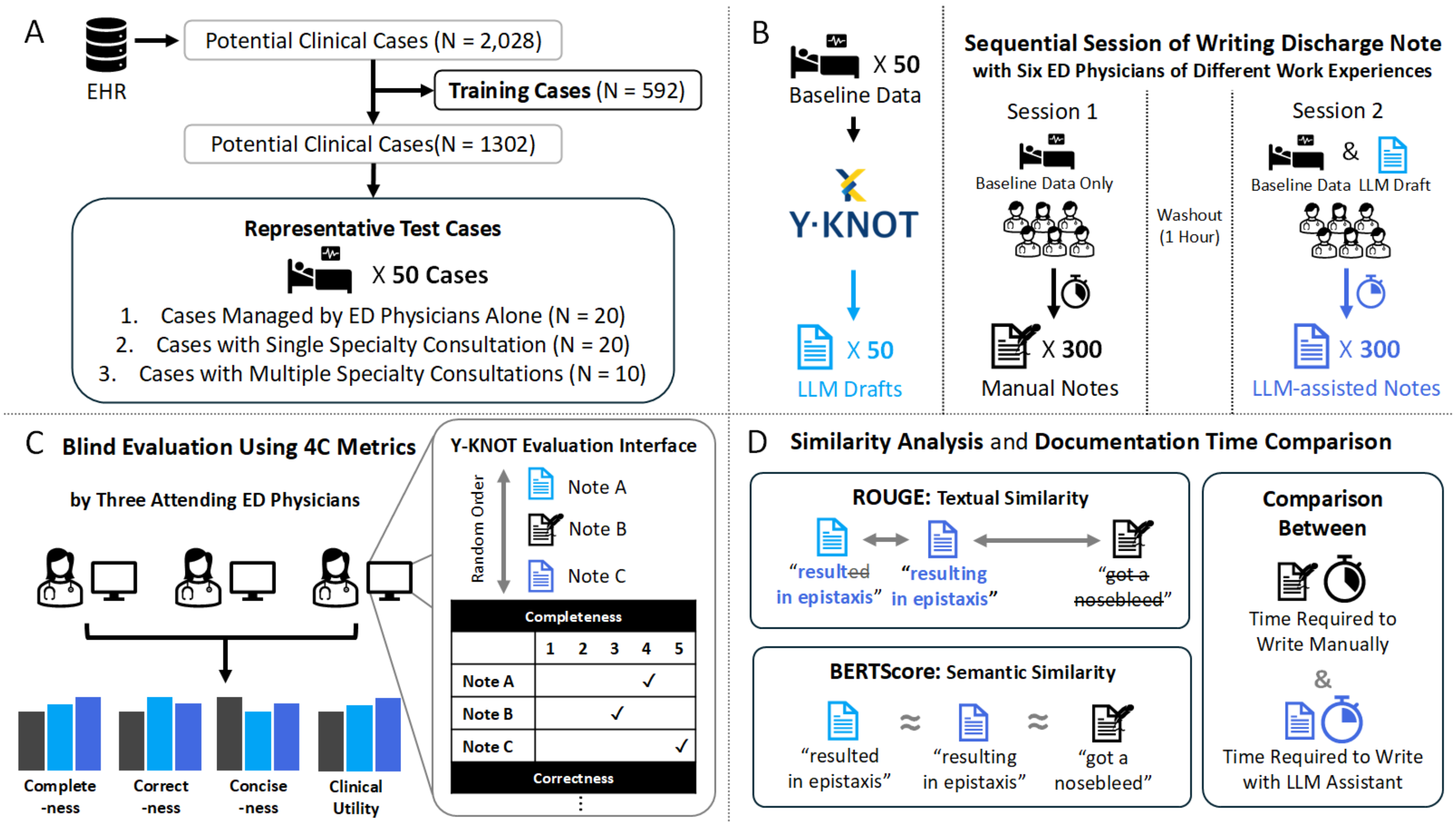

(A) Data selection involved 2,028 ED cases, from which 592 cases were curated for training and 50 representative test cases (20 without consultations, 20 with single consultations, 10 with multiple consultations) were selected.
(B) Sequential sessions with six emergency physicians included manual note-writing in Session 1 and LLM-assisted note-writing in Session 2, using 50 baseline data sets and LLM drafts. Writing time was recorded across both sessions.
(C) Blinded clinical evaluation of 300 note sets (Manual, LLM Draft, LLM-Assisted) by three attending physicians was conducted using 4Cs metrics (Completeness, Correctness, Conciseness, Clinical Utility) on a 5-point Likert scale.
(D) Textual (ROUGE) and semantic (BERTScore) similarity analyses compared LLM-assisted notes with manual notes and LLM drafts. Writing time reduction between manual and LLM-assisted notes was also analyzed.

© 2025 Song JW et al. *JAMA Network Open*.

eFigure 2. Screenshot of Our Institution's EHR User Interface

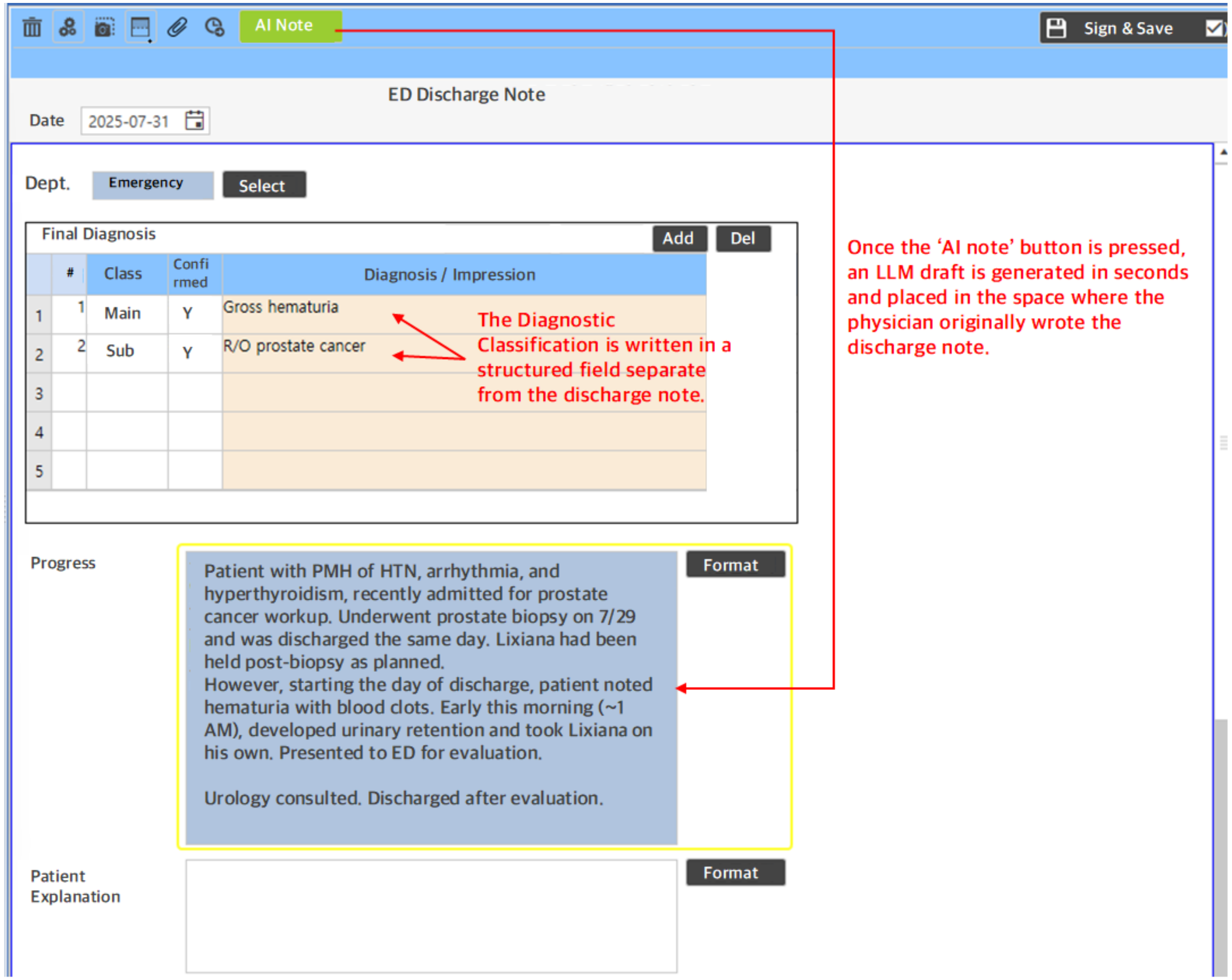

The Final Diagnosis section captures diagnoses in a structured field, separate from the narrative note. The AI Note control triggers the integrated Y-KNOT-EDN service. The lower Progress area displays the generated/editable LLM draft. Original Korean labels and the LLM draft are translated to English. As shown, the system is currently in full use with Y-KNOT-EDN fully integrated into the live EHR environment.

© 2025 Song JW et al. *JAMA Network Open*.

eFigure 3. Example of the Customized Interface for Blind Evaluation of 3 Kinds of Note Using the 4Cs Metrics

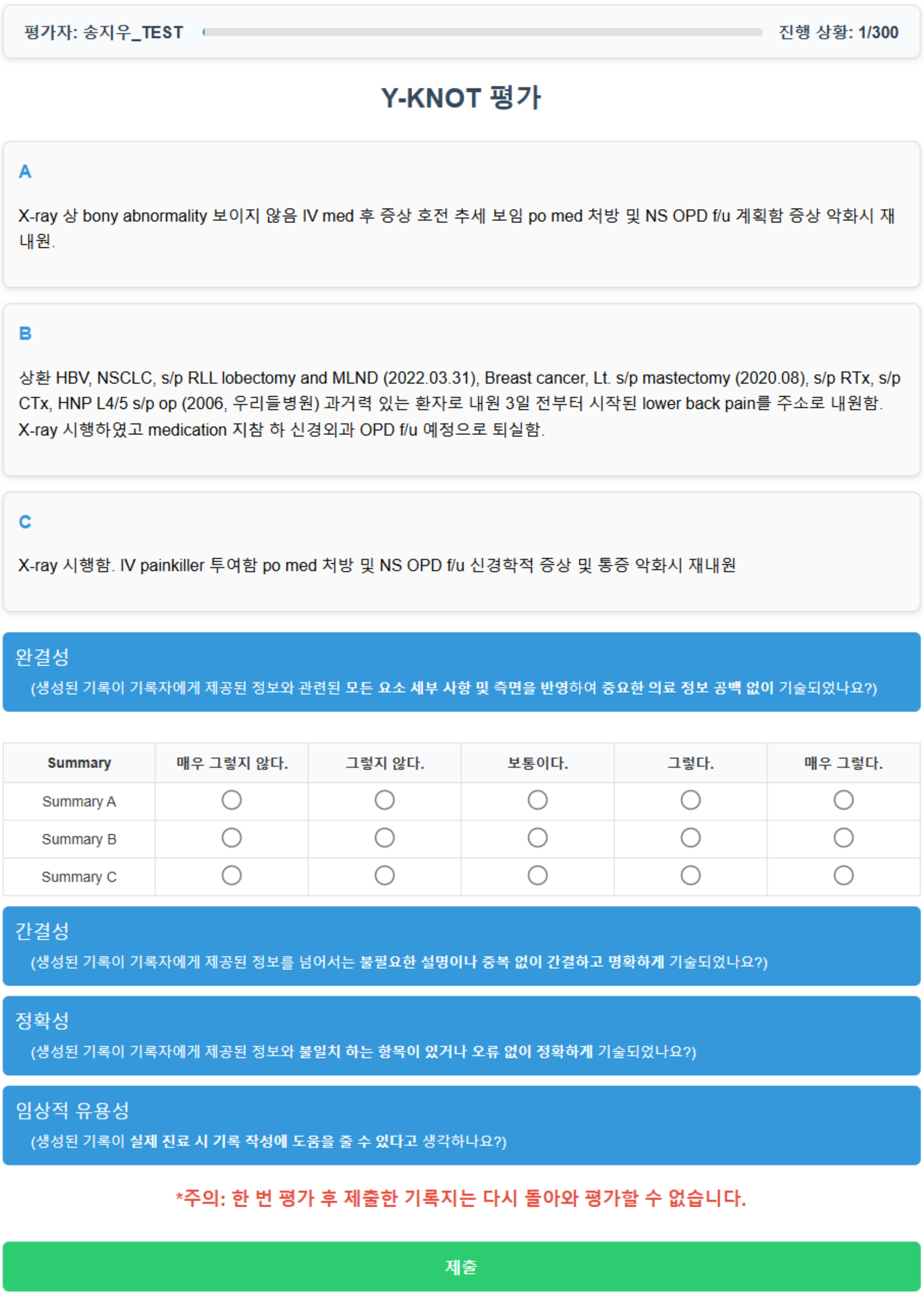

An example of the user interface created in Korean, which displays the manual note, LLM draft, and LLM-assisted note in a random order for blind assessment, is a screenshot of a website created by one of our research team's Research Assistants. For each set, there were three sections labeled A, B, and C, each of which was randomly assigned to one of the three note types (manual note, LLM draft, or LLM-assisted note), thus ensuring that evaluators do not know which note corresponds to which category. In the center, there were four collapsible blue sections for rating completeness, correctness, conciseness, and clinical utility (the "4Cs"). Each section could be expanded or collapsed with a click, and the definitions for the 4Cs were provided in parentheses for clarity. The three summaries—A, B, and C—wee scored blindly on a 1–5 Likert scale under each metric. Moreover, three hundred sets of the manual note, LLM draft, and LLM-assisted note were shown in block divided into six blocks and randomized. Within each block, the cases from 1st to 50th are shuffled randomly, and the identity of the physician who wrote each note is mixed up.

© 2025 Song JW et al. *JAMA Network Open*.

eFigure 4. Pairwise Rater-Agreement Heat Maps for the 3 Physician Evaluators

(A)

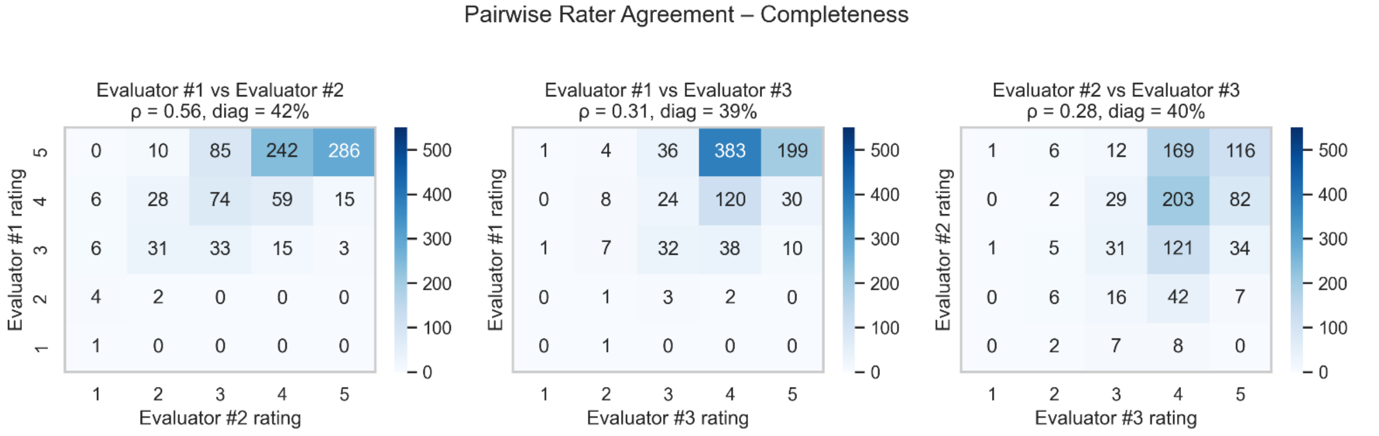

(B)

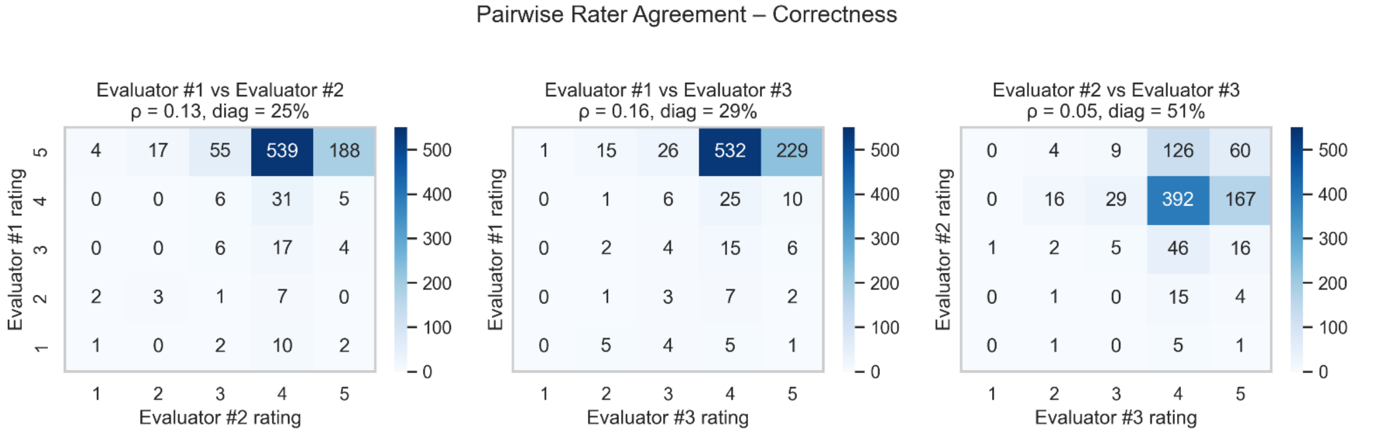

(C)

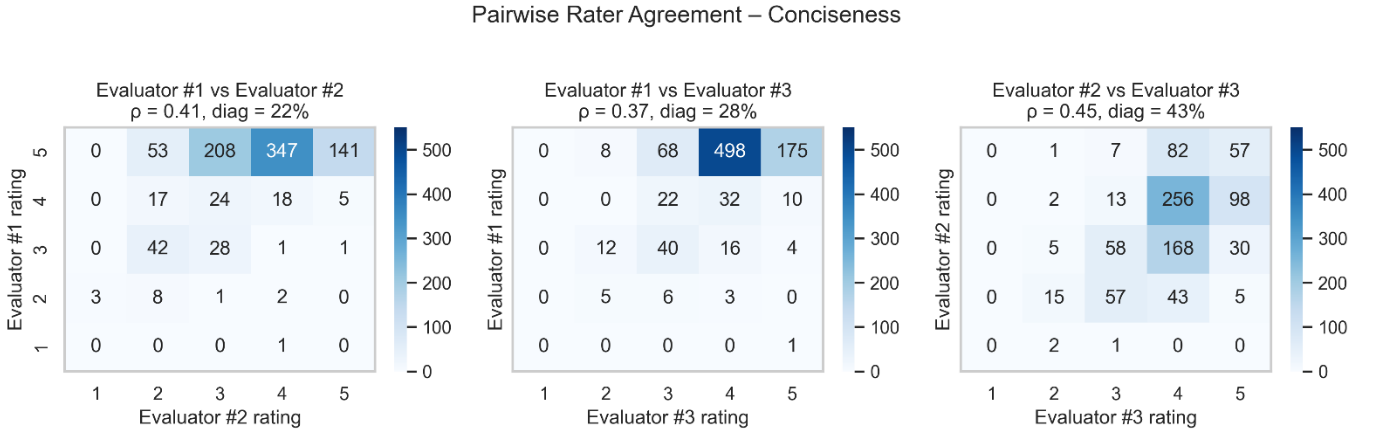

(D)

© 2025 Song JW et al. *JAMA Network Open*.

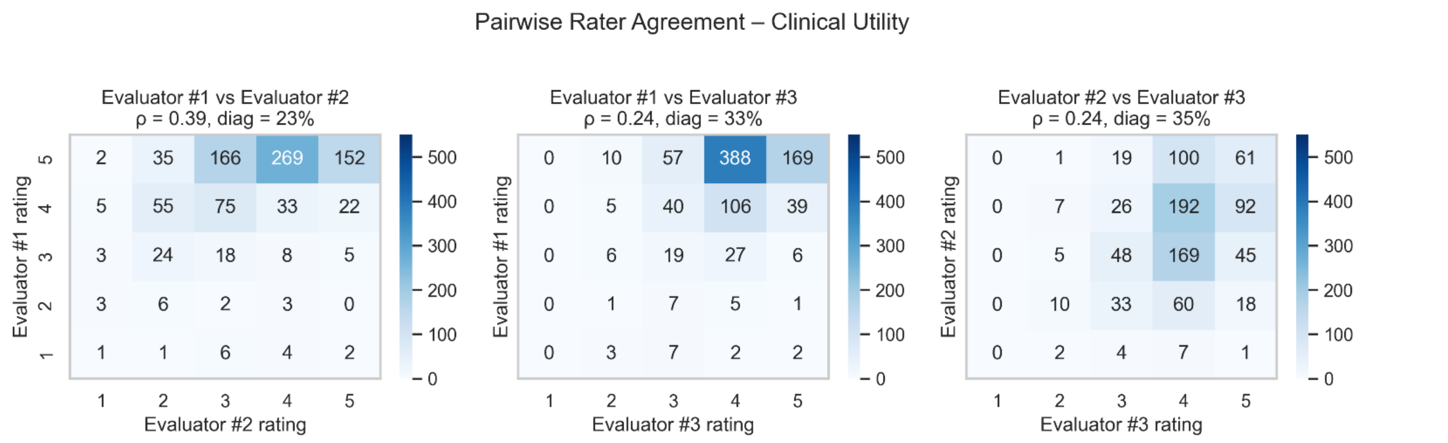

Each panel contains three 5 × 5 matrices comparing the two-way ratings of every evaluator pair (Evaluator #1 vs #2, #1 vs #3, #2 vs #3). The axes give the 5-point Likert scores assigned by the respective evaluators; cell colour intensity denotes the number of discharge summaries receiving that exact score combination (common scale across all panels, darker = more summaries). The inset text above each matrix reports Spearman's rank correlation (ρ) between the two sets of scores and the percentage of summaries that fall on the main diagonal (diag = exact agreement). Together, the heatmaps visualize both the strength and pattern of inter-rater agreement within each evaluation domain.

eFigure 5. Subgroup Results Categorized by Complexity of Consultation

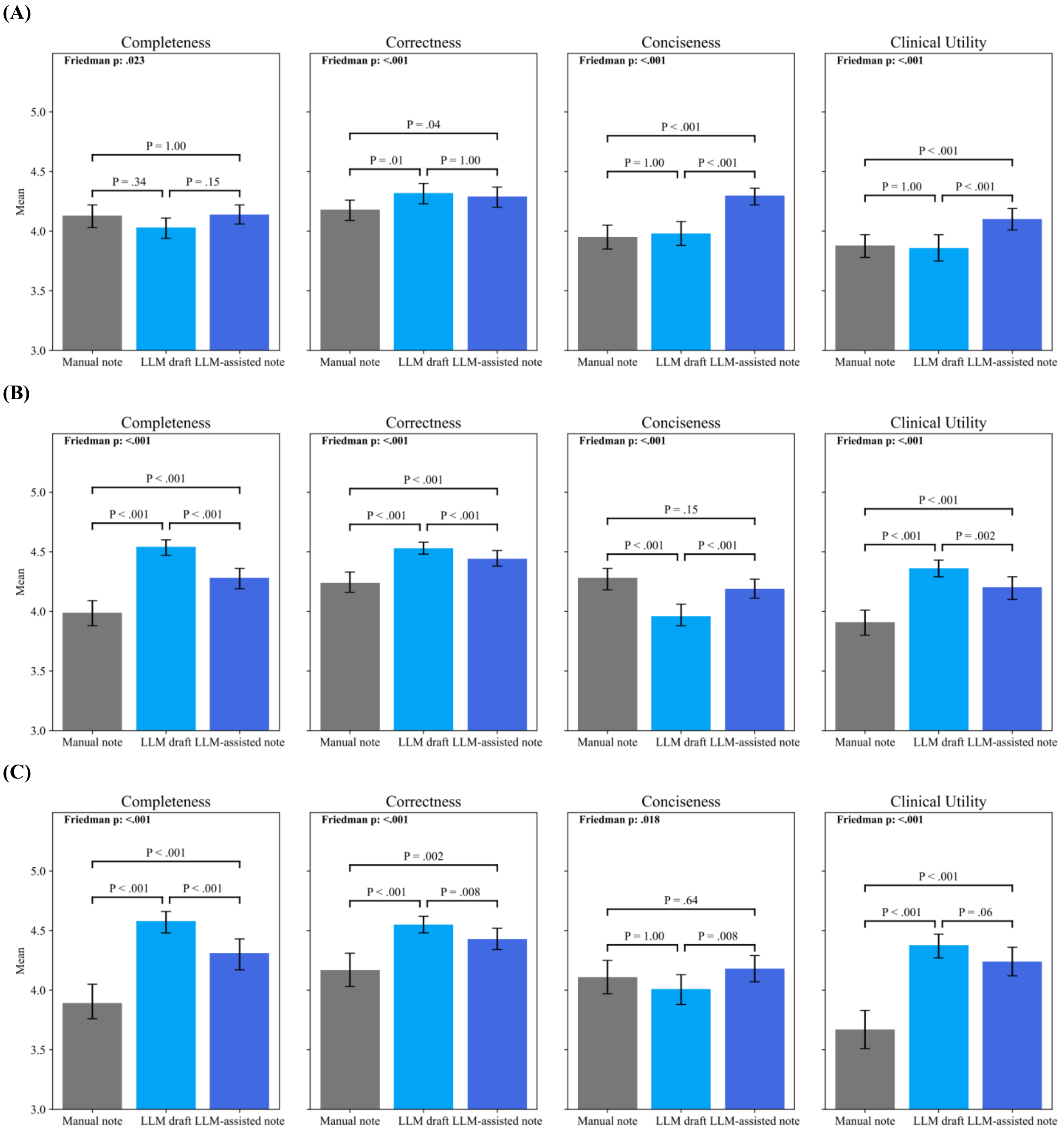

Bars depict mean scores with 95 % bootstrap confidence intervals; exact Bonferroni-adjusted P values are annotated between paired comparisons. Y-axis is truncated to the clinically relevant range (3 – 5).

(A) In cases managed only by emergency physicians, the LLM-assisted note was significantly more concise than both the manual and LLM draft notes, with comparable completeness and correctness to the LLM draft. Clinical utility was highest for the LLM-assisted note.

(B) For single specialty consultation, the LLM draft was the most complete and correct, while the LLM-assisted note was more concise than LLM draft and clinically useful than the manual note.

(C) In multiple consultation cases, the LLM draft also scored highest in completeness and correctness, with the LLM-assisted note being more concise than LLM draft and clinically useful than the manual note.

© 2025 Song JW et al. *JAMA Network Open*.

eFigure 6. Expected Writing-Time Ratio (LLM-Assisted or Manual) Estimated From a Crossed Random-Effects Log-Normal Mixed Model

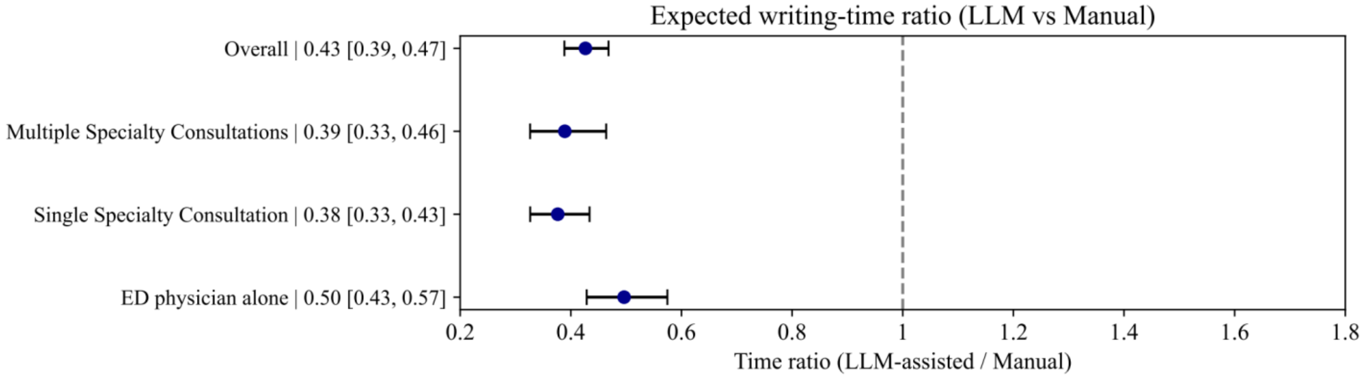

Forest-plot showing the multiplicative change in note-writing time when using the LLM assistant, overall and stratified by case-complexity. Points denote the estimated time-ratio (RR < 1 indicates faster documentation with the LLM); horizontal bars are Wald 95 % confidence intervals. All models adjust simultaneously for complexity while including random intercepts for patient-case and physician to account for the crossed data structure.

eTable 1. Definition of 4C Metrics for Qualitative Evaluation of Discharge Notes

**Metric**	**Definition**
Completeness	Does the generated record reflect all relevant elements, details, and aspects of the provided information without omitting any critical medical details?
Correctness	Is the generated record free from discrepancies or errors, accurately matching the information given?
Conciseness	Is the generated record clear and succinct, without unnecessary or redundant explanations beyond what was provided?
Clinical utility	How useful would the generated record be for actual clinical documentation?

eTable 2. Main Results of Entire Notes

Metric	Type of Information	Type of Note	Mean (95% CI)	SD	Likert					Friedman/ Wilcoxon P	Hedge's g (95% CI)	HL (95% CI)
					1	2	3	4	5			
Completeness	Descriptive Statistics	Manual Note	4.03 (3.96 ~ 4.09)	0.95	13	49	172	334	332			
		LLM Draft	4.34 (4.29 ~ 4.39)	0.74	1	13	103	344	439			
		LLM-Assisted Note	4.23 (4.17 ~ 4.28)	0.85	6	36	100	363	395			
	Statistical Comparison	Friedman								<0.001		
		Wilcoxon (LLM Draft vs Manual Note)								<0.001	-0.26 (-0.33 ~ -0.2)	0 (0 ~ 0)
		Wilcoxon (LLM Draft vs LLM-Assisted Note)								0.001	0.12 (0.06 ~ 0.18)	0 (0 ~ 0)
		Wilcoxon (Manual Note vs LLM-Assisted Note)								<0.001	-0.22 (-0.28 ~ -0.15)	0 (0 ~ 0)
Correctness	Descriptive Statistics	Manual Note	4.20 (4.14 ~ 4.25)	0.85	11	35	81	407	366			
		LLM Draft	4.45 (4.41 ~ 4.49)	0.65	5	9	21	407	458			
		LLM-Assisted Note	4.38 (4.33 ~ 4.42)	0.71	7	13	38	416	426			
	Statistical Comparison	Friedman								<0.001		
		Wilcoxon (LLM Draft vs Manual Note)								<0.001	-0.27 (-0.33 ~ -0.21)	0 (0 ~ 0)
		Wilcoxon (LLM Draft vs LLM-Assisted Note)								<0.001	0.12 (0.06 ~ 0.18)	0 (0 ~ 0)
		Wilcoxon (Manual Note vs LLM-Assisted Note)								<0.001	-0.20 (-0.27 ~ -0.14)	0 (0 ~ 0)
Conciseness	Descriptive Statistics	Manual Note	4.11 (4.05 ~ 4.17)	0.95	1	70	147	291	391			
		LLM Draft	3.98 (3.91 ~ 4.04)	0.95	3	68	191	324	314			
		LLM-Assisted Note	4.23 (4.18 ~ 4.28)	0.78	0	21	131	367	381			
	Statistical Comparison	Friedman								<0.001		
		Wilcoxon (LLM Draft vs Manual Note)								0.004	0.12 (0.06 ~ 0.19)	0 (0 ~ 0)
		Wilcoxon (LLM Draft vs LLM-Assisted Note)								<0.001	-0.31 (-0.38 ~ -0.25)	0 (0 ~ 0)
		Wilcoxon (Manual Note vs LLM-Assisted Note)								<0.001	-0.13 (-0.19 ~ -0.06)	0 (0 ~ 0)
Clinical Utility	Descriptive Statistics	Manual Note	3.85 (3.78 ~ 3.91)	1.01	13	81	218	305	283			
		LLM Draft	4.16 (4.11 ~ 4.21)	0.87	6	39	128	356	371			
		LLM-Assisted Note	4.17 (4.11 ~ 4.23)	0.88	9	40	109	374	368			
	Statistical Comparison	Friedman								<0.001		
		Wilcoxon (LLM Draft vs Manual Note)								<0.001	-0.26 (-0.33 ~ -0.2)	0 (0 ~ 0)
		Wilcoxon (LLM Draft vs LLM-Assisted Note)								1.000	-0.01 (-0.07 ~ 0.05)	0 (0 ~ 0)
		Wilcoxon (Manual Note vs LLM-Assisted Note)								<0.001	-0.29 (-0.36 ~ -0.23)	0 (0 ~ 0)

A total of 900 evaluation scores, corresponding to each type of note, were recorded in the Likert score columns. These scores were provided by three evaluators, each assessing 300 notes. The p-value column shows the result of Friedman test conducted on the three note types. If significant differences emerged in the test, pairwise comparisons were performed using the Wilcoxon signed-rank test.

© 2025 Song JW et al. *JAMA Network Open*.

eTable 3. Subgroup Results Categorized by Consultation Complexity

(A) Managed Only by Emergency Physicians

Metric	Type of Information	Type of Note	Mean (95% CI)	SD	Likert					Friedman/ Wilcoxon P	Hedge's g (95% CI)	HL (95% CI)
					1	2	3	4	5			
Completeness	Descriptive Statistics	Manual Note	4.13 (4.03 ~ 4.22)	0.89	1	16	67	128	148			
		LLM Draft	4.03 (3.94 ~ 4.11)	0.79	1	11	69	175	104			
		LLM-Assisted Note	4.14 (4.06 ~ 4.22)	0.83	2	15	44	169	130			
	Statistical Comparison	Friedman								0.023		
		Wilcoxon (LLM Draft vs Manual Note)								0.344	0.08 (-0.03 ~ 0.19)	0 (0 ~ 0)
		Wilcoxon (LLM Draft vs LLM-Assisted Note)								0.151	-0.11 (-0.23 ~ -0.01)	0 (0 ~ 0)
		Wilcoxon (Manual Note vs LLM-Assisted Note)								1.000	-0.01 (-0.12 ~ 0.09)	0 (0 ~ 0)
Correctness	Descriptive Statistics	Manual Note	4.18 (4.09 ~ 4.26)	0.80	3	10	41	173	133			
		LLM Draft	4.32 (4.23 ~ 4.40)	0.80	5	9	18	163	165			
		LLM-Assisted Note	4.29 (4.20 ~ 4.37)	0.78	4	8	23	170	155			
	Statistical Comparison	Friedman								<0.001		
		Wilcoxon (LLM Draft vs Manual Note)								0.012	-0.14 (-0.25 ~ -0.04)	0 (0 ~ 0)
		Wilcoxon (LLM Draft vs LLM-Assisted Note)								1.000	0.04 (-0.07 ~ 0.13)	0 (0 ~ 0)
		Wilcoxon (Manual Note vs LLM-Assisted Note)								0.035	-0.13 (-0.24 ~ -0.03)	0 (0 ~ 0)
Conciseness	Descriptive Statistics	Manual Note	3.95 (3.85 ~ 4.05)	1	1	38	67	125	129			
		LLM Draft	3.98 (3.88 ~ 4.08)	1.02	3	38	59	125	135			
		LLM-Assisted Note	4.30 (4.22 ~ 4.36)	0.73	0	8	33	163	156			
	Statistical Comparison	Friedman								<0.001		
		Wilcoxon (LLM Draft vs Manual Note)								1.000	-0.02 (-0.12 ~ 0.08)	0 (0 ~ 0)
		Wilcoxon (LLM Draft vs LLM-Assisted Note)								<0.001	-0.34 (-0.43 ~ -0.25)	0 (0 ~ 0)
		Wilcoxon (Manual Note vs LLM-Assisted Note)								<0.001	-0.37 (-0.47 ~ -0.28)	0 (0 ~ 0)
Clinical Utility	Descriptive Statistics	Manual Note	3.88 (3.78 ~ 3.97)	0.94	2	25	97	128	108			
		LLM Draft	3.86 (3.75 ~ 3.97)	1.01	6	35	72	138	109			
		LLM-Assisted Note	4.10 (4.01 ~ 4.19)	0.89	5	15	51	156	133			
	Statistical Comparison	Friedman								<0.001		
		Wilcoxon (LLM Draft vs Manual Note)								1.000	0.01 (-0.09 ~ 0.11)	0 (0 ~ 0)
		Wilcoxon (LLM Draft vs LLM-Assisted Note)								<0.001	-0.22 (-0.32 ~ -0.12)	0 (0 ~ 0)
		Wilcoxon (Manual Note vs LLM-Assisted Note)								<0.001	-0.21 (-0.31 ~ -0.1)	0 (0 ~ 0)

© 2025 Song JW et al. *JAMA Network Open*.

(B) Single specialty consultation

Metric	Type of Information	Type of Note	Mean (95% CI)	SD	Likert					Friedman/ Wilcoxon P	Hedge's g (95% CI)	HL (95% CI)
					1	2	3	4	5			
Completeness	Descriptive Statistics	Manual Note	3.99 (3.88 ~ 4.09)	0.95	7	15	76	139	123			
		LLM Draft	4.54 (4.47 ~ 4.60)	0.64	0	1	25	114	220			
		LLM-Assisted Note	4.28 (4.19 ~ 4.36)	0.85	3	13	38	133	173			
	Statistical Comparison	Friedman								<0.001		
		Wilcoxon (LLM Draft vs Manual Note)								<0.001	-0.49 (-0.60 ~ -0.39)	0 (-0.5 ~ 0)
		Wilcoxon (LLM Draft vs LLM-Assisted Note)								<0.001	0.3 (0.22 ~ 0.38)	0 (0 ~ 0)
		Wilcoxon (Manual Note vs LLM-Assisted Note)								<0.001	-0.34 (-0.45 ~ -0.24)	0 (0 ~ 0)
Correctness	Descriptive Statistics	Manual Note	4.24 (4.16 ~ 4.33)	0.85	4	14	30	154	158			
		LLM Draft	4.53 (4.48 ~ 4.58)	0.51	0	0	2	165	193			
		LLM-Assisted Note	4.44 (4.38 ~ 4.51)	0.64	2	4	5	170	179			
	Statistical Comparison	Friedman								<0.001		
		Wilcoxon (LLM Draft vs Manual Note)								<0.001	-0.36 (-0.42 ~ -0.30)	0 (0 ~ 0)
		Wilcoxon (LLM Draft vs LLM-Assisted Note)								<0.001	0.19 (0.13 ~ 0.25)	0 (0 ~ 0)
		Wilcoxon (Manual Note vs LLM-Assisted Note)								<0.001	-0.25 (-0.34 ~ -0.15)	0 (0 ~ 0)
Conciseness	Descriptive Statistics	Manual Note	4.28 (4.18 ~ 4.36)	0.85	0	15	50	116	179			
		LLM Draft	3.96 (3.88 ~ 4.06)	0.91	0	20	95	124	121			
		LLM-Assisted Note	4.19 (4.11 ~ 4.27)	0.83	0	10	65	132	153			
	Statistical Comparison	Friedman								<0.001		
		Wilcoxon (LLM Draft vs Manual Note)								<0.001	0.30 (0.19 ~ 0.41)	0 (0 ~ 0)
		Wilcoxon (LLM Draft vs LLM-Assisted Note)								<0.001	-0.32 (-0.42 ~ -0.22)	0 (0 ~ 0)
		Wilcoxon (Manual Note vs LLM-Assisted Note)								0.147	0.10 (0 ~ 0.2)	0 (0 ~ 0)
Clinical Utility	Descriptive Statistics	Manual Note	3.91 (3.80 ~ 4.01)	1.01	5	30	80	122	123			
		LLM Draft	4.36 (4.29 ~ 4.43)	0.70	0	2	40	144	174			
		LLM-Assisted Note	4.20 (4.10 ~ 4.29)	0.91	4	19	38	139	160			
	Statistical Comparison	Friedman								<0.001		
		Wilcoxon (LLM Draft vs Manual Note)								<0.001	-0.41 (-0.51 ~ -0.31)	0 (0 ~ 0)
		Wilcoxon (LLM Draft vs LLM-Assisted Note)								0.002	0.18 (0.09 ~ 0.27)	0 (0 ~ 0)
		Wilcoxon (Manual Note vs LLM-Assisted Note)								<0.001	-0.28 (-0.39 ~ -0.18)	0 (0 ~ 0)

© 2025 Song JW et al. *JAMA Network Open*.

(C) Multiple specialty consultations

Metric	Type of Information	Type of Note	Mean (95% CI)	SD	Likert					Friedman/ Wilcoxon P	Hedge's g (95% CI)	HL (95% CI)
					1	2	3	4	5			
Completeness	Descriptive Statistics	Manual Note	3.89 (3.76 ~ 4.05)	1.07	5	18	29	67	61			
		LLM Draft	4.58 (4.48 ~ 4.66)	0.62	0	1	9	55	115			
		LLM-Assisted Note	4.31 (4.17 ~ 4.43)	0.87	1	8	18	61	92			
	Statistical Comparison	Friedman								<0.001		
		Wilcoxon (LLM Draft vs Manual Note)								<0.001	-0.57 (-0.70 ~ -0.45)	0 (-1 ~ 0)
		Wilcoxon (LLM Draft vs LLM-Assisted Note)								<0.001	0.31 (0.19 ~ 0.44)	0 (0 ~ 0)
		Wilcoxon (Manual Note vs LLM-Assisted Note)								<0.001	-0.39 (-0.52 ~ -0.26)	0 (0 ~ 0)
Correctness	Descriptive Statistics	Manual Note	4.17 (4.03 ~ 4.31)	0.94	4	11	10	80	75			
		LLM Draft	4.55 (4.48 ~ 4.62)	0.51	0	0	1	79	100			
		LLM-Assisted Note	4.43 (4.34 ~ 4.52)	0.68	1	1	10	76	92			
	Statistical Comparison	Friedman								<0.001		
		Wilcoxon (LLM Draft vs Manual Note)								<0.001	-0.40 (-0.48 ~ -0.32)	0 (0 ~ 0)
		Wilcoxon (LLM Draft vs LLM-Assisted Note)								0.008	0.22 (0.13 ~ 0.31)	0 (0 ~ 0)
		Wilcoxon (Manual Note vs LLM-Assisted Note)								0.002	-0.26 (-0.39 ~ -0.13)	0 (0 ~ 0)
Conciseness	Descriptive Statistics	Manual Note	4.11 (3.97 ~ 4.25)	1.00	0	17	30	50	83			
		LLM Draft	4.01 (3.88 ~ 4.13)	0.87	0	10	37	75	58			
		LLM-Assisted Note	4.18 (4.07 ~ 4.29)	0.79	0	3	33	72	72			
	Statistical Comparison	Friedman								0.018		
		Wilcoxon (LLM Draft vs Manual Note)								1.000	0.09 (-0.07 ~ 0.23)	0 (0 ~ 0)
		Wilcoxon (LLM Draft vs LLM-Assisted Note)								0.008	-0.25 (-0.38 ~ -0.11)	0 (0 ~ 0)
		Wilcoxon (Manual Note vs LLM-Assisted Note)								0.636	-0.07 (-0.22 ~ 0.08)	0 (0 ~ 0)
Clinical Utility	Descriptive Statistics	Manual Note	3.67 (3.51 ~ 3.83)	1.14	6	26	41	55	52			
		LLM Draft	4.38 (4.27 ~ 4.47)	0.69	0	2	16	74	88			
		LLM-Assisted Note	4.24 (4.12 ~ 4.36)	0.78	0	6	20	79	75			
	Statistical Comparison	Friedman								<0.001		
		Wilcoxon (LLM Draft vs Manual Note)								<0.001	-0.56 (-0.71 ~ -0.42)	-0.5 (-1 ~ 0)
		Wilcoxon (LLM Draft vs LLM-Assisted Note)								0.057	0.18 (0.04 ~ 0.31)	0 (0 ~ 0)
		Wilcoxon (Manual Note vs LLM-Assisted Note)								<0.001	-0.49 (-0.65 ~ -0.36)	0 (-1 ~ 0)

The evaluation scores of (A) 20 cases managed by ED physicians alone, (B) 20 cases with single specialty consultation involved and (C) 10 cases with multiple specialty consultation involved were recorded in the Likert score columns in each table, respectively. These scores were provided by three evaluators, each assessing 120, 120 and 60 notes from six ED physicians. The p-value column shows the result of Friedman test conducted on the three note types. If significant differences emerged in the test, pairwise comparisons were performed using the Wilcoxon signed-rank test.

© 2025 Song JW et al. *JAMA Network Open*.

eTable 4. Sensitivity Analysis Results

Metric	Type of Information	Type of Note	Mean (95% CI)	SD	Likert					Friedman/ Wilcoxon P	Hedge's g (95% CI)	HL (95% CI)
					1	2	3	4	5			
Completeness	Descriptive Statistics	Manual Note	4.20 (4.05 ~ 4.35)	0.90	2	4	25	50	69			
		LLM Draft	4.39 (4.25 ~ 4.53)	0.84	1	3	20	38	88			
		LLM-Assisted Note	4.43 (4.30 ~ 4.55)	0.76	0	3	16	45	86			
	Statistical Comparison	Friedman								0.026		
		Wilcoxon (LLM Draft vs Manual Note)								0.245	-0.15 (-0.30 ~ 0.01)	0 (-0.5 ~ 0)
		Wilcoxon (LLM Draft vs LLM-Assisted Note)								1.000	-0.04 (-0.20 ~ 0.13)	0 (0 ~ 0)
		Wilcoxon (Manual Note vs LLM-Assisted Note)								0.063	-0.20 (-0.36 ~ -0.06)	0 (0 ~ 0)
Correctness	Descriptive Statistics	Manual Note	4.69 (4.56 ~ 4.79)	0.74	1	5	4	20	120			
		LLM Draft	4.79 (4.69 ~ 4.88)	0.62	1	3	1	17	128			
		LLM-Assisted Note	4.75 (4.63 ~ 4.85)	0.69	2	2	3	18	125			
	Statistical Comparison	Friedman								0.037		
		Wilcoxon (LLM Draft vs Manual Note)								0.401	-0.12 (-0.28 ~ 0.04)	0 (0 ~ 0)
		Wilcoxon (LLM Draft vs LLM-Assisted Note)								1.000	0.05 (-0.10 ~ 0.22)	0 (0 ~ 0)
		Wilcoxon (Manual Note vs LLM-Assisted Note)								0.955	-0.07 (-0.24 ~ 0.08)	0 (0 ~ 0)
Conciseness	Descriptive Statistics	Manual Note	4.27 (4.10 ~ 4.44)	1.01	0	13	22	26	89			
		LLM Draft	4.07 (3.89 ~ 4.24)	1.04	1	13	31	34	71			
		LLM-Assisted Note	4.36 (4.21 ~ 4.49)	0.83	0	5	19	43	83			
	Statistical Comparison	Friedman								0.013		
		Wilcoxon (LLM Draft vs Manual Note)								0.252	0.15 (-0.02 ~ 0.30)	0 (0 ~ 0)
		Wilcoxon (LLM Draft vs LLM-Assisted Note)								0.002	-0.29 (-0.44 ~ -0.15)	0 (0 ~ 0)
		Wilcoxon (Manual Note vs LLM-Assisted Note)								1.000	-0.07 (-0.23 ~ 0.08)	0 (0 ~ 0)
Clinical Utility	Descriptive Statistics	Manual Note	4.17 (4.04 ~ 4.31)	0.93	1	2	41	32	74			
		LLM Draft	4.35 (4.21 ~ 4.48)	0.87	2	4	15	48	81			
		LLM-Assisted Note	4.43 (4.30 ~ 4.55)	0.80	1	2	17	42	88			
	Statistical Comparison	Friedman								0.025		
		Wilcoxon (LLM Draft vs Manual Note)								0.263	-0.15 (-0.31 ~ 0.02)	0 (0 ~ 0)
		Wilcoxon (LLM Draft vs LLM-Assisted Note)								1.000	-0.08 (-0.23 ~ 0.07)	0 (0 ~ 0)
		Wilcoxon (Manual Note vs LLM-Assisted Note)								0.025	-0.22 (-0.37 ~ -0.06)	0 (0 ~ 0)

The evaluation scores of the first 50 cases per evaluator (totaling 150 cases) were recorded in the Likert score columns in each table, respectively. The p-value column shows the result of Friedman test conducted on the three note types. If significant differences emerged in the test, pairwise comparisons were performed using the Wilcoxon signed-rank test.

© 2025 Song JW et al. *JAMA Network Open*.

eTable 5. Textual and Semantic Similarity Captured by ROUGE and BERTScore

The LLM-Assisted Note as Reference Text

Metric	Physician	LLM-Assisted Note and		Type of Statistical Test	p-value
		Manual Note	LLM Draft		
ROUGE-1	Overall	0.718 (0.695 ~ 0.743)	0.836 (0.811 ~ 0.86)	Wilcoxon signed-rank	<0.001
	1	0.629 (0.563 ~ 0.690)	0.563 (0.510 ~ 0.614)	Wilcoxon signed-rank	0.159
	2	0.726 (0.660 ~ 0.782)	0.936 (0.885 ~ 0.974)	Wilcoxon signed-rank	<0.001
	3	0.780 (0.731 ~ 0.827)	0.851 (0.809 ~ 0.894)	Wilcoxon signed-rank	0.003
	4	0.749 (0.692 ~ 0.804)	0.844 (0.770 ~ 0.909)	Wilcoxon signed-rank	0.016
	5	0.680 (0.623 ~ 0.734)	0.912 (0.871 ~ 0.946)	Wilcoxon signed-rank	<0.001
	6	0.746 (0.682 ~ 0.804)	0.912 (0.886 ~ 0.939)	Wilcoxon signed-rank	<0.001
ROUGE-2	Overall	0.587 (0.555 ~ 0.617)	0.764 (0.732 ~ 0.796)	Wilcoxon signed-rank	<0.001
	1	0.459 (0.387 ~ 0.529)	0.457 (0.399 ~ 0.511)	Paired t-test	0.976
	2	0.622 (0.537 ~ 0.695)	0.906 (0.841 ~ 0.96)	Wilcoxon signed-rank	<0.001
	3	0.638 (0.568 ~ 0.709)	0.728 (0.647 ~ 0.803)	Wilcoxon signed-rank	0.006
	4	0.642 (0.566 ~ 0.716)	0.804 (0.720 ~ 0.884)	Wilcoxon signed-rank	0.005
	5	0.523 (0.453 ~ 0.593)	0.849 (0.781 ~ 0.904)	Wilcoxon signed-rank	<0.001
	6	0.636 (0.557 ~ 0.706)	0.841 (0.783 ~ 0.894)	Wilcoxon signed-rank	<0.001
ROUGE-L	Overall	0.691 (0.666 ~ 0.715)	0.828 (0.801 ~ 0.852)	Wilcoxon signed-rank	<0.001
	1	0.586 (0.519 ~ 0.646)	0.544 (0.491 ~ 0.598)	Paired t-test	0.397
	2	0.694 (0.627 ~ 0.755)	0.934 (0.885 ~ 0.973)	Wilcoxon signed-rank	<0.001
	3	0.741 (0.687 ~ 0.794)	0.830 (0.784 ~ 0.875)	Wilcoxon signed-rank	<0.001
	4	0.727 (0.666 ~ 0.787)	0.842 (0.768 ~ 0.909)	Wilcoxon signed-rank	0.009
	5	0.663 (0.607 ~ 0.717)	0.909 (0.868 ~ 0.944)	Wilcoxon signed-rank	<0.001
	6	0.736 (0.673 ~ 0.792)	0.908 (0.88 ~ 0.935)	Wilcoxon signed-rank	<0.001
BERTScore	Overall	0.932 (0.928 ~ 0.936)	0.965 (0.96 ~ 0.97)	Wilcoxon signed-rank	<0.001
	1	0.904 (0.894 ~ 0.914)	0.891 (0.883 ~ 0.898)	Paired t-test	0.077
	2	0.935 (0.927 ~ 0.944)	0.990 (0.984 ~ 0.995)	Wilcoxon signed-rank	<0.001
	3	0.946 (0.94 ~ 0.951)	0.974 (0.968 ~ 0.98)	Wilcoxon signed-rank	<0.001
	4	0.936 (0.927 ~ 0.945)	0.974 (0.963 ~ 0.984)	Wilcoxon signed-rank	<0.001
	5	0.930 (0.925 ~ 0.935)	0.979 (0.973 ~ 0.984)	Wilcoxon signed-rank	<0.001
	6	0.942 (0.936 ~ 0.948)	0.984 (0.98 ~ 0.988)	Wilcoxon signed-rank	<0.001

Values are reported as the mean ± 95 % bootstrap confidence interval. For the scores of ROUGE and BERTScore, f-measure and f1-score are used, respectively. Since the discharge notes are written primarily in Korean, with some English, we used a multilingual tokenizer (XLM-RoBERTa) for BERTScore.

© 2025 Song JW et al. *JAMA Network Open*.

eTable 6. Median Time (Seconds) Required to Write the Manual Note and the LLM Assisted Note: Overall Results and Breakdown by Consultation Complexity and Individual Physician

	**Manual Note Median (95% CI)**	**LLM Assisted Note Median (95% CI)**	**H-L (95% CI)**	**p-value**
Overall	69.5 (65.5-78)	32 (29.5-36)	35 (29.5-41.5)	<0.001
Consultation				
No	69 (58-78.5)	33 (28-39)	24 (20-32)	<0.001
Single	67.5 (60-83)	30 (26-34)	43 (35-52)	<0.001
Multiple	80.5 (68.5-92.5)	38.5 (28-46)	48.5 (31-55)	<0.001
Physician				
1	66 (55-75.5)	41 (35.5-50)	19.5 (13-29)	<0.001
2	60 (51-75.5)	13.5 (9-24.5)	43 (34-51.5)	<0.001
3	68.5 (61-89)	46 (39-56)	20.5 (19-27)	<0.001
4	46 (42-59.5)	28 (24-35.5)	16.5 (8.5-31)	<0.001
5	87 (80.5-107.5)	30 (24.5-36)	54 (42-72)	<0.001
6	90.5 (80-113.5)	33.5 (29-40)	55.5 (48-72)	<0.001

eTable 7. User Experience Survey Regarding 12 Aspects of Y-KNOT

A brief user experience survey about the utility of Y-KNOT system was done to subject ED physicians after finishing session 2. The survey asked about 12 aspects of Y-KNOT: consistency, coherence, relevance, correctness, fluency, similarity with actual summary, time-saving, helpfulness, degree of revision required, intention to use, intention to delegate and patient safety. All questions were answered with a Likert scale ranging from 1 to 5.

		**Likert rating, No.**				
**Criteria**	**Mean (SD)**	**1**	**2**	**3**	**4**	**5**
Consistency	4.2 (0.8)	0	0	1	3	2
Coherence	4.3 (0.5)	0	0	0	4	2
Fluency	3.7 (1.5)	1	0	1	2	2
Relevance	4.3 (0.5)	0	0	0	4	2
Correctness	3.7 (0.8)	0	1	0	5	0
Time-Saving	4.3 (0.5)	0	0	0	4	2
Helpfulness	4.3 (0.8)	0	0	1	2	3
Patient Safety	2.5 (0.5)	0	3	3	0	0
Similarity	3.3 (0.8)	0	1	2	3	0
Revision Required[a]	2.5 (0.5)	0	3	3	0	0
Intention to Use	3.8 (0.4)	0	0	1	5	0
Intention to Delegate	3.3 (0.5)	0	0	4	2	0

[a]Note: Lower "Revision Required" is better.

© 2025 Song JW et al. *JAMA Network Open*.

eTable 8. Real Examples of Omissions and Confabulation Identified in the 50-Case Audit of LLM Drafts

Case#	Relevant snippet of input (truth)	What the LLM draft did	Error category	Severity	Clinician concordance/correction (n/6)
**3**	CRF (Cardiology): reason for referral included INR prolongation + pancytopenia (MDS f/u)	Summarized INR 7.34 but omitted pancytopenia	Omission	Minor	Manual note also omitted: 1/6
**9**	ED IR: Headache with vomiting;<br>CRF (Neurology): Headache for r/o meningitis	Selected “Headache” as the chief complaint and omitted vomiting	Omission	Minor	Manual note also omitted: 4/6
**11**	CRF (Neurosurgery): notes patient is already followed up in neurosurgery clinic	Draft omitted prior NS clinic follow-up	Omission	Minor	Manual note also omitted: 4/6
**12**	CRF (Cardiology) explicitly says “return to ED if discomfort”	Draft omitted the CRF’s explicit return-precaution sentence	Omission	Minor	Manual note also omitted: 4/6
**22**	CRF (Hospitalist): AKI resolved while hydrating for rhabdomyolysis (CK↑); continued hydration plan 120 cc/h and outpatient CK f/u	Draft summarized rhabdomyolysis and hydration plan but did not state “AKI resolved.”	Omission	Minor	Manual note also omitted: 4/6
**45**	ED IR: assault with occipital blow by bottle (wound description present)	Draft omitted the mechanism of injury	Omission	Minor	Manual note also omitted: 4/6
**50**	Orders/Rx pane included elbow X-ray, sedation, and suture, but did not include splint or dressing orders	Draft confabulated “Splint apply” and “Dressing” despite no corresponding orders	Confabulation	Major	Corrected in LLM-assisted note: 5/6

Omissions observed here did not change clinical context or threaten safety; the single hallucination warrants caution. ED IR = Emergency Department Initial Record; CRF = Specialty Consultation Request Form; Rx = prescription/ordering pane.

© 2025 Song JW et al. *JAMA Network Open*.
