# Supplementary material for: Large Language Model Assistant for Emergency Department Discharge Documentation
Source: JAMA Netw Open. 2025 Oct 21;8(10):e2538427. doi: 10.1001/jamanetworkopen.2025.38427 (PMC12541540; doi:10.1001/jamanetworkopen.2025.38427)
Supplement: Supplement 2. — Data Sharing Statement [file jamanetwopen-e2538427-s002.pdf]

Data Sharing Statement

Song. Large Language Model Assistant for Emergency Department Discharge Documentation. *JAMA Netw Open*. Published October 21, 2025. doi:10.1001/jamanetworkopen.2025.38427

Data
Data available: No

Additional Information
Explanation for why data not available: The study's underlying data include deidentified free-text clinical notes (manual, LLM draft, and LLM-assisted versions) and blinded evaluation scores from three emergency attendings. While free-text clinical notes contain sensitive clinical information with a risk of re-identification, they will not be made publicly available. Inquiries should be directed to the designated corresponding author (SCY).
